# Interferon regulatory factor 4 modulates epigenetic silencing and cancer‐critical pathways in melanoma cells

**DOI:** 10.1002/1878-0261.13672

**Published:** 2024-06-16

**Authors:** Ulduz Sobhiafshar, Betül Çakici, Erdem Yilmaz, Nalan Yildiz Ayhan, Laila Hedaya, Mustafa Can Ayhan, Cansu Yerinde, Yasemin Begüm Alankuş, H. Kübra Gürkaşlar, Elif Nur Firat‐Karalar, N. C. Tolga Emre

**Affiliations:** ^1^ Department of Molecular Biology and Genetics Boğaziçi University Istanbul Turkey; ^2^ Department of Molecular Biology and Genetics Koç University Istanbul Turkey; ^3^ Center for Life Sciences and Technologies Boğaziçi University Istanbul Turkey

**Keywords:** DNA methylation, epi‐drugs, epigenetic silencing, histone methylation, IRF4, melanoma

## Abstract

Interferon regulatory factor 4 (IRF4) was initially identified as a key controller in lymphocyte differentiation and function, and subsequently as a dependency factor and therapy target in lymphocyte‐derived cancers. In melanocytes, IRF4 takes part in pigmentation. Although genetic studies have implicated IRF4 in melanoma, how IRF4 functions in melanoma cells has remained largely elusive. Here, we confirmed prevalent IRF4 expression in melanoma and showed that high expression is linked to dependency in cells and mortality in patients. Analysis of genes activated by IRF4 uncovered, as a novel target category, epigenetic silencing factors involved in DNA methylation (DNMT1, DNMT3B, UHRF1) and histone H3K27 methylation (EZH2). Consequently, we show that IRF4 controls the expression of tumour suppressor genes known to be silenced by these epigenetic modifications, for instance cyclin‐dependent kinase inhibitors CDKN1A and CDKN1B, the PI3–AKT pathway regulator PTEN, and primary cilium components. Furthermore, IRF4 modulates activity of key downstream oncogenic pathways, such as WNT/β‐catenin and AKT, impacting cell proliferation and survival. Accordingly, IRF4 modifies the effectiveness of pertinent epigenetic drugs on melanoma cells, a finding that encourages further studies towards therapeutic targeting of IRF4 in melanoma.

## Graphical abstract

In this study, we showed that high interferon regulatory factor 4 (IRF4) expression is linked to dependency in melanoma cells and mortality in patients. Transcriptomic analysis uncovered epigenetic silencing factors as a novel target category. IRF4 consequently controls the expression of tumour suppressor genes known to be silenced by these epigenetic factors, and the activity of key downstream oncogenic pathways.

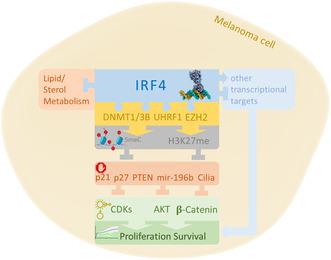

**Abbreviations**
5‐Aza: 5‐azacytidine
5‐mC: 5‐methylcytosine
AXIN2: axin 2
CCLE: Cancer Cell Line Encyclopedia
CDK: cyclin‐dependent kinases
ChIP: chromatin immunoprecipitation
CKIs: cyclin‐dependent kinase inhibitors
DAC: decitabine
DEGs: differentially expressed genes
DepMap: Dependedency Map
DLBCL: diffuse large B‐cell lymphoma
DNMT1: DNA methyltransferase 1
DNMT3B: DNA methyltransferase 3B
Dox: doxycycline
ENCODE: The Encyclopedia of DNA Elements
EZH2: enhancer of zeste 2 polycomb repressive complex 2 subunit
FPKM: log‐scale fragments per kilobases per million mapped reads
GO: gene ontology
GSK‐3b: glycogen synthase kinase‐3 beta
H3K27me3: histone H3 lysine 27 tri‐methylation
H3K4me1: H3 lysine 4 monomethylation
ICSAT: interferon consensus sequence binding protein for activated T cells
IRF4: interferon regulatory factor 4
KO: knockout
LSIRF: lymphocyte‐specific interferon regulatory factor
MAPK: mitogen activated protein kinase
MET: hepatocyte growth factor receptor (HGF receptor)
MITF: melanocyte inducing transcription factor
MSRE‐qPCR: methylation sensitive restriction enzyme digestion analysis
OE: overexpressing
pAKT: Ser473‐phosphorylated AKT
PD‐L1: programmed death‐ligand 1
PI3K: phosphoinositide 3‐kinase
PRC2: polycomb repressive complex 2
pS6: Ser235/236‐phosphorylated S6 protein
PTEN: phosphatase and tensin homologue
RICTOR: RPTOR independent companion of MTOR complex 2
RICTOR: RPTOR independent companion of MTOR complex 2
RRID: Research Resource Identifier
SKCM: skin cutaneous melanoma
TCGA: The Cancer Genome Atlas
TSGs: tumour suppressor genes
Tyr: tyrosinase
UHRF1: ubiquitin like with phd and ring finger domains 1
UVM: uveal melanoma
WNT3aCM: WNT3a‐conditioned medium

## 1 Introduction

IRF4 belongs to the interferon regulatory factor (IRF) family of transcriptional regulators [1] and is also known as NF‐EM5, Pip, LSIRF, ICSAT, or MUM1. It was discovered as a factor involved in immunoglobin expression in B‐cells and was later shown to perform critical roles in the development and effector functions of various immune cell subsets. IRF4 expression was shown to be essentially regulated by developmental and mitogenic signals, in contrast to most other IRF family members, which are regulated by interferon signalling [2, 3].

Early studies implicated IRF4 in malignancies [2, 4]. Through drop‐out RNAi library screens, we identified IRF4 as a critical factor for cancers derived from mature B‐cells, i.e., multiple myeloma [5] and aggressive diffuse large B‐cell lymphoma [6]. In these cancers, mutations at *IRF4* locus are not common; however, IRF4 is highly expressed. Significantly, only a ~ 50% reduction in IRF4 levels is sufficient to manifest proliferation and survival defects in malignant cells, suggesting their dependence on IRF4 [5, 6]. IRF4 was found to be similarly critical for a variety of other lymphoma types with high IRF4 expression [7, 8, 9, 10]. Remarkably, in supporting proliferation and survival, IRF4 can engage distinct genes and pathways in different cancer types [11], such as MYC [5, 8] or NF‐κB [6]. As a testament to IRF4's immune cancer‐critical role, immunomodulatory imid drugs (IMiDs) used in cancer therapy, such as lenalidomide, provide therapeutic benefits largely through reducing IRF4 expression [12, 13].

IRF4 also functions outside of the immune system, for instance in adipose tissue [14] and in the pigment‐producing cells, melanocytes [15, 16]. Interestingly, several genetic studies have associated a single nucleotide polymorphism (SNP; rs12203592) in the fourth intron of *IRF4* with various pigmentation phenotypes, such as hair and eye colour, nevus count, and skin response to sunlight [17, 18, 19]. Subsequently, the SNP‐containing locus was shown to harbour a melanocyte lineage‐specific enhancer for *IRF4*, where the polymorphism affects the binding of the transcriptional regulators TFAP2A and MITF [15], and with other factors, modulates enhancer‐promoter contact [20] for IRF4 expression. In turn, IRF4 regulates the expression of key melanin production enzymes [15], and ultraviolet light responses in melanocytic cells [21].

Previous correlative evidence implicated IRF4 also in melanoma, the most lethal form of skin cancer, derived from melanocytes [22]. Early studies demonstrated IRF4 expression in a melanoma cell line [23] and in tumours of melanoma patients [24, 25], to the extent that IRF4 staining was proposed as a sensitive and specific marker for melanoma [25] similar to lymphoid malignancies [26]. In addition, numerous association studies have linked the *IRF4* fourth intron germline SNP to melanomas [27, 28, 29] and to an IRF4‐mediated gene regulatory program [30]. Furthermore, in line with fourth intron enhancer‐mediated regulation of IRF4 expression by MITF in the melanocytic lineage, IRF4 expression was observed to be higher in melanoma subpopulations and cell states associated with high MITF expression, such as the proliferative state [31], melanocytic and transitionary subtypes [32], melanocytic state/subtype [33, 34]. However, if and how IRF4 is causally involved in melanoma has largely remained an open question.

In this study, we first verified that IRF4 expression is widespread in melanoma. More importantly, IRF4 is critical for the fitness of IRF4‐expressing melanoma cells, and high IRF4 expression is associated with poor patient survival. We show for the first time that IRF4 is an upstream regulator of melanoma‐critical gene‐repressive epigenetic factors and their epigenetic marks, i.e., 5‐cytosine methylation of DNA and lysine 27 methylation of histone H3. Accordingly, we demonstrate that IRF4 regulates key tumour suppressor genes and oncogenic pathways downstream of these epigenetic effects. We further show that IRF4 modulates melanoma cell responses to the relevant methylation inhibitor drugs.

## 2 Materials and methods

### 2.1 Cell culture

Cell lines, SKMEL28 (RRID: CVCL_0526, gift from Eiríkur Steingrímsson, University of Iceland), G361, A375 (RRID: CVCL_1220, and CVCL_0132, gift from Tolga Sütlü, Acibadem University), UACC62, Malme3M and MEL‐ST (RRID: CVCL_1780, CVCL_1438, gift from María S. Soengas, CNIO) were used. HEK293FT cell line was used for lentiviral production (RRID: CVCL_6911, gift from Ferruh Özcan, Gebze Technical University). Cells were cultured in high‐Glucose DMEM with l‐glutamine (Gibco, Thermo Fisher Scientific, Waltham, MA, USA, cat. #41966029) supplemented with 10% FBS (Gibco, Thermo Fisher Scientific, cat. #10270106 or Hyclone, Cytiva, Marlborough, MA, USA, cat. #SV30160.03), 1% penicillin/streptomycin (Pan Biotech, Aidenbach, Germany, cat. #P06‐07100), and 1% non‐essential amino acids (Pan Biotech, cat. #p08‐32100). Cultures were maintained in an incubator with 5% CO_2_ and at 37 °C. Cells were passaged every 3–4 days. For IRF4 genotyping of the cell lines at 4th intron SNP (rs12203592), PCR and Sanger sequencing were used, as previously described [15]. Sanger DNA sequencing was performed at Macrogen Inc (Seoul, South Korea). For conditional (inducible) heterologous expression of IRF4 or Cas9, stably transduced cell lines were treated with 500–750 ng·mL^−1^ of doxycycline (Sigma, Merck, Darmstadt, Germany, cat. #D5207). All cell line identities were authenticated by the PowerPlex16HS assay:15 Autosomal Loci, X/Y kit (Promega, Madison, WI, USA) at the University of Arizona Genetics Core. All cell lines were periodically tested for *Mycoplasma* contamination by PCR.

### 2.2 Plasmids and cloning

For shRNA knockdown experiments, shLuc (targeting firefly luciferase gene, as control), shRPS13, shIRF4‐1, or shIRF4‐2 (all 3 targeting 3′ UTRs of the corresponding genes) were designed and cloned into KH1 lentiviral vector using XmaI (NEB, Ipswich, MA, USA, cat. #R0180S) and XbaI (NEB, R0145S) restriction enzymes briefly as follows: individual RNAi trigger sequences and their complementary strands were ordered from Macrogen Inc. as MOPS‐purified long oligos (for sequences, refer to Table S1). Upon annealing of complementary strands (which created XmaI and XbaI restriction site compatible overhangs) and 5′ end phosphorylation using T4 PNK (NEB, cat. #M0201S), oligo duplexes were ligated into gel‐purified and digested KH1 vector. Post‐ligation, recombinant vectors were transformed into the Stbl3 strain of *Escherichia coli*.

For stable ectopic expression of IRF4 in cell lines, a doxycycline‐inducible lentiviral pINDUCER20 gateway vector was obtained from Addgene, Watertown, MA, USA (RRID: Addgene_44012; a gift from Stephen Elledge). pDONR221‐IRF4 was obtained from DNASU (HsCD00040446, DNASU). IRF4 cDNA was subcloned from pDONR221 to pINDUCER20 using Gateway LR Clonase II (Thermo Fisher Scientific, cat. #11791043) as described by the manufacturer.

For inducible expression of shIRF4 in cell lines, a doxycycline inducible lentiviral vector pINDUCER10 was obtained from Addgene (RRID: Addgene_44011; a gift from Stephen Elledge). shIRF4 sequences used in KH1 vector were cloned into pINCUCER10 vector as described [35].

For CRISPR/Cas9‐mediated knockout of IRF4 in melanoma cell lines, pCW‐Cas9‐blast (RRID: Addgene_83481, a gift from Mohan Babu) and pLKO5.sgRNA.EFS.GFP (RRID: Addgene_57822, a gift from Benjamin Ebert) plasmids were used. Oligos for guide RNA generation targeting IRF4 (i.e., sgIRF4 KO1 and sgIRF4 KO3) were designed and cloned essentially as described [36] (for sequences, refer to Table S1).

### 2.3 Reagents and antibodies

DNA methylation inhibitors, 5‐Azacytidine (Aza; cat. #S1782), Decitabine (Dac; cat. #S1200‐10 mg), and the EZH2 inhibitor, EPZ‐6438 (cat. #S7128), were all purchased from SelleckChem, Houston, TX, USA. All three drugs were dissolved in DMSO. In all experiments, the stock solution was 750×–1000× of the final concentration needed in the assay. Cells were treated daily with 5‐Aza and Dac for 3–5 days in the 0.062–1 μm concentration range. EPZ‐6438 treatment was applied every other day for 6–8 days in the 0.5–8 μm range.

Information about antibodies used in this study is provided in Table S2.

### 2.4 Lentiviral transduction and generation of stable cell lines

Lentivirus was produced by transfecting HEK293FT cells with the lentiviral vector of interest and second‐generation packaging vectors psPAX2 (RRID: Addgene_12260, a gift from Didier Trono) and pCMV‐VSV‐G (Addgene #8454, a gift from Robert Weinberg) with a modified protocol of calcium phosphate transfection method [37]. Briefly, 10 μg of lentiviral vector, mixed with 4 μg of pCMV‐VSV‐G, 7.5 μg of psPAX2 packaging plasmids, 62.5 μL of 2 m CaCl_2_ and nuclease‐free water mixed well. Five hundred microlitre of HEPES‐buffer saline (Alfa Aesar, Haverhill, MA, USA, cat. #J62623) was added dropwise and mixed to create bubbles. Virus‐containing medium (supernatant) was collected 48 h post‐transfection. After passing through a 0.45 μm filter, it was aliquoted and kept at −80 °C freezer.

Lentiviral transduction was performed by seeding cells the day before transduction at 20–25% confluency. The next day, thawed lentivirus‐containing medium was added together with polybrene‐supplemented (8 μg·mL^−1^, Sigma‐Aldrich, cat. #H9268) medium to cells, and 8 h post‐transduction, the medium was changed. For selection and various assays, cells were seeded 8 h post‐transduction. The pINDUCER20‐transduced cell lines were selected with 1 mg·mL^−1^ G418 (SantaCruz, Dallas, TX, USA, cat. #SC‐29065A) treatment, whereas pINDUCER10‐transduced cell lines were selected with 2 μg·mL^−1^ of puromycin (SantaCruz, cat. #SC‐108071) treatment.

In CRISPR‐Cas9 vector‐transduced cells, the selection was done with 4 μg·mL^−1^ Blasticidin (AppliChem, Darmstadt, Germany, cat. #A3784.0010). Antibiotic selection of the cells was continued until the control untransduced cells were all killed. During this derivation process, doxycycline was withheld (i.e., Cas9 expression kept off). To obtain IRF4‐knockout, doxycycline was introduced to the growth medium of the cells transduced with sgRNA vector for Cas9 expression. Depletion of IRF4 protein levels is typically first detected 12–14 days after doxycycline induction (i.e., near or complete disappearance of IRF4 signal in immunoblot). Phenotypic analyses (e.g., cell death) are accordingly performed within the third week of this time frame.

### 2.5 RNA isolation, cDNA synthesis, and qPCR analysis

According to the manufacturer's instructions, total RNA was extracted from cells using NucleoZol (Macharey‐Nagel, cat. #740404.2). After elution in nuclease‐free water and absorbance measurement for quantification and purity determination with Nanodrop ND‐1000 (Thermo Fisher Scientific), cDNA synthesis was carried out using iScript™ cDNA Synthesis Kit (BioRad, Hercules, CA, USA, cat. #1708891) with 750 ng RNA according to the manufacturer's protocol. Semiquantitative PCR (qPCR) was then carried out using SYBR Green master mix (Ampliqon, Odense, Denmark, cat. #A323406) according to the instructions of the manufacturer, starting with 2.5 μL of 1 : 25 diluted cDNA and 0.3 μm of each forward and reverse gene‐specific exon‐spanning primers, using PikoReal instrument (Thermo Scientific). Relative expression values were calculated using the ΔΔCt method [38] and normalised to corresponding signals from a housekeeping gene (RPS28 or PGK1) from three technical replicates. The oligonucleotide primers used for RT‐qPCR are listed in Table S3.

### 2.6 Western blotting

Subconfluent cells were trypsinised, collected, and washed with cold PBS twice and resuspended in RIPA buffer (150 mm NaCl, 1% NP‐40, 0.5% Na‐deoxycholate, 0.1% SDS, 50 mm Tris pH8) supplemented with protease inhibitor cocktail (Roche, cat. #11873580001) and phosphatase inhibitor cocktail (Roche, Basel, Switzerland, cat. #4906845001). After a 30‐min incubation on ice, lysates were sonicated at 40% power with a 15 s ON/45 s OFF pulse for 3 min in a Q800R1 sonicator (Qsonica, Newtown, CT, USA). Subsequently, lysates were centrifuged at 12 000 ***g*** for 10 min and kept in a −20 °C freezer. The protein concentration of the lysates was measured with BCA Assay (Pierce, Thermo Fisher Scientific, cat. #23227). For sub‐cellular fractionation, PBS‐washed cells were scraped and mixed with cytoplasmic extraction buffer (25 mm Tris–HCl pH 8, 20 mm NaCl, 2 mm EDTA, 0.5% Tween 20). After 15 min of incubation on ice, samples were centrifuged at 6000 ***g*** for 3 min at 4 °C. The cytoplasmic fraction, the supernatant, was separated. Then the pellet, consisting of nuclei, was resuspended in nuclear lysis buffer (25 mm Tris–HCl pH 7.4, 150 mm NaCl, 1 mm EDTA, 1% NP40 substitute, 5% Glycerol) and incubated for 40 min on ice with a long vortex every 10 min. Post centrifugation at 14 000 ***g*** for 10 min, the supernatant was collected as the nuclear fraction.

Lysates were loaded and separated on 10%, 12%, or 15% SDS‐acrylamide gels. After transferring proteins to Immobilon PVDF 0.22 μm (Millipore, Merck, cat. #ISEQ00010) or Hybond 0.45 μm PVDF (Amersham, Cytiva, UK, cat. #10600023) membranes, they were blocked with 5% milk in TBS/0.1% Tween20 for 1 h at room temperature. Primary antibodies in 5% BSA (BioShop Canada, Burlington, ON, Canada, cat. #ALB001.100) were incubated with corresponding membranes overnight at +4 °C. After primary antibody incubation and subsequent TBS‐T wash (3 × 5 min) steps, membranes were incubated with species‐appropriate HRP‐conjugated secondary antibodies 1 : 3000 dilution for 1 h at room temperature. The chemiluminescence signal from HRP was detected using WsesternBright ECL or Sirius HRP substrates (Advansta, San Jose, CA, USA, cat. #K‐12045‐D50, #K‐12043‐D10, respectively) on a G‐box chemi: XRQ instrument (RRID: SCR_015770, Syngene, Cambridge, UK).

### 2.7 Chromatin immunoprecipitation

For anti‐IRF4 chromatin immunoprecipitation (ChIP), 20–40 million cells were crosslinked with 1% formaldehyde (Sigma‐Aldrich, cat. #15512) for 10 min at room temperature. Then the cross‐linking reaction was quenched by incubation with 0.125 m glycine (Sigma‐Aldrich, cat. #33226) for 5 min. Cells were washed twice with PBS, resuspended in ChIP lysis buffer (50 mm HEPES, 150 mm NaCl, 1% Triton X‐100, 0.1% Na‐deoxycholate, 1 mm EDTA) supplemented with SDS (0.25% final) and protease inhibitor cocktail (Roche, cat. #11873580001). After 45 min incubation on ice, lysed cells were sonicated (each for 1 min. at 80% duty cycle and 85% power output) with an MS72 tip‐fitted Bandelin Sonoplus HD2070 sonicator or in Q800R1 water‐bath sonicator (Qsonica) at 70% power, 15 : 45 s ON/OFF cycle, and a total time of 15 min. Following sonication, lysates were cleared by centrifugation (14 000 rpm/18 700 ***g*** for 10 min at 4 °C). Soluble chromatin lysate derived from ~ 5 million cells after three‐fold dilution with ChIP lysis buffer (with protease inhibitor cocktail) was incubated with 50 μL of mixed protein G and protein A DynaBeads magnetic beads (Thermo Fisher Scientific, cat. #10003D, #10004D) prebound with 5 μg anti‐iRF4 antibody (Santa Cruz, sc‐6059, or made‐to‐order goat polyclonal anti‐IRF4 antibody, Proteogenix Inc., Schiltigheim, France) or normal goat IgG (Santa Cruz, cat. #sc‐2028) overnight at 4 °C. The next day, bead‐bound immune complexes were washed for 1 min at room temperature twice with each of the following buffers: ChIP Lysis Buffer, High‐salt ChIP Lysis Buffer with 500 mm NaCl, and TE Buffer. Then bead‐bound immune complexes and corresponding sonicated lysates (to be used as “input control”) were boiled for 10 min in TE, which was followed by Rnase A treatment (Sigma, cat. #R4875; 0.1 μg·μL^−1^ final) for 45 min at 39 °C (input controls only), and afterward, Proteinase K treatment (Jena Bioscience, Jena, Germany, cat. #EN‐178L; 200 ng·μL^−1^ final) for 30 min at 55 °C ensued. Following enzymatic treatments, samples were boiled again for 10 min, and DNA residing in the supernatant was purified using silica‐based spin columns (Macherey Nagel, cat. #740609, or Zymo Research, Irvine, CA, USA, cat. #D5205). Purified and enriched DNA and input‐control DNA were then diluted 10‐ to 100‐fold and subjected to real‐time PCR amplification in triplicates with region‐specific primer pairs (for sequences, refer to Table S3). The resulting qPCR data from each ChIP were then analysed with the ΔΔCt method and normalised to corresponding input DNA samples' data.

### 2.8 Identification of ChIP‐qPCR candidate regions

Available published ENCODE Dnase‐seq of IRF4‐expressing melanoma cell lines (SKMEL‐5, Colo829) and the immortalised melanocyte Melano cell line was loaded on to UCSC Genome Browser (version: hg19; [39]). JASPAR [40] transcription factor binding site consensus motifs for the IRF family transcription factors were also added. Candidate IRF4‐regulated regions were designated as those regions that overlapped in Dnase‐seq signals and contained binding motifs for the IRF family transcription factors. The negative control was chosen from an intergenic region with no prediction or evidence of any IRF family member binding motifs. Primers were designed spanning these selected regions to be used in ChIP‐qPCR.

### 2.9 Enzyme‐linked immunosorbent assay for 5‐methyl‐cytosine DNA (5‐mC ELISA)

One million cells were lysed with cell lysis buffer (100 mm NaCl, 10 mm Tris pH 8.0, 25 mm EDTA pH 8.0, 0.5% SDS, 0.1 mg·mL^−1^ Proteinase K) overnight at 55 °C on the thermomixer, and genomic DNA (gDNA) extraction was performed with gDNA isolation kit (Zymo Research, cat. #D3024).

One hundred nanograms of denatured gDNA was prepared and coated the wells in a 96‐well plate in duplicates. 5‐mC ELISA was carried out using Zymo 5‐mC ELISA kit according to the manufacturer's protocol (Zymo Research cat. #D5326). ELISA results were read with a plate reader at 430 nm, at three different time points of 15, 35, and 55 min, alongside a dilution series of kit‐supplied standard 5‐mC DNA mixtures. The values at the timepoint with the best standard curve (i.e., highest correlation coefficient) were used for further analysis in MS Excel. Graphs were generated with prism 8, Graphpad, Boston, MA, USA (RRID: SCR_002798).

### 2.10 Methylation sensitive restriction enzyme digestion analysis (MSRE‐qPCR)

MSRE‐qPCR was carried out essentially as described [41]. Briefly, 500 ng of gDNA was incubated with a mixture of four different restriction enzymes: AciI (NEB cat. #R0551S), HpaII (NEB, cat. #R0171S), HinP1I (NEB, cat. #R0124S) HpyCH4IV (NEB, cat. #R0619S) according to manufacturer's protocol. Digested DNA and control DNA were diluted 1 : 10–1 : 20. qPCR was carried out similarly to qPCR conditions mentioned in the ChIP‐qPCR section. MSRE‐qPCR primer design was carried out on the promoter region of the candidate genes with two criteria: (a) Inverse correlation between CpG methylation at the gene promoter loci and gene expression data obtained from TCGA and CCLE databases for each target gene; (b) at least 3 of 4 restriction enzyme sites are present in the candidate region.

### 2.11 Luciferase reporter assay

100 000 cells were seeded in 24‐well plates. The following day, TOPFLASH (3× TCF/LEF binding elements, Sigma‐Aldrich, cat. #21‐170) and pRL‐SV40‐Renilla (Promega, cat. #E2231) plasmids (both gifts from Necla Birgül Iyison, Boğaziçi University) were transfected using K2 transfection reagent according to their recommended protocol (Biontex, Munich, Germany, cat. #T060‐1.0). Twenty‐four hours post‐transfection, cells were treated with LiCl (final concentration 10 mm), Wnt3a‐conditioned medium, or pIRES‐GFP (Clonetech, cat. #6029‐1)‐transfected conditioned medium as control (1 : 1 mixed with complete DMEM). Forty‐eight hours after transfection, cells were lysed, and luciferase assay was carried out using Luc‐Pair™ duo‐luciferase assay kit (Genecopoeia, Rockville, MD, cat. #LF001).

To prepare conditioned media, L929 cells were transfected with an pLNCX‐wnt3a vector (gift from Necla Birgül Iyison, Boğaziçi University) or pIRES‐GFP vector as control, using K2 transfection reagent as described by the manufacturer. Forty‐eight and seventy‐two hours after transfection, conditioned medium (CM) was collected and stored at −20 °C for future use.

### 2.12 RNA‐seq and Bioinformatic analysis

Total RNA was isolated from three independent replicates, each of shLuc‐ and shIRF4‐transduced SKMEL28 and SKMEL5 cells on day 3 post‐transduction. Following integrity control of RNA with Bioanalyzer 2100 (Agilent, Santa Clara, CA, USA)RNA samples were sent to UCLA Genomics Core (University of California, LA). Library preparation was carried out with Illumina TruSeq RNA sample preparation kit according to the manufacturer's protocol. Samples were sequenced as single‐end 50 bp reads on a HiSeq2000 instrument (Illumina, San Diego, CA, USA) for SKMEL28 and a HiSeq2500 instrument (Illumina) for SKMEL5 samples. After data quality control with FASTQC (RRID: SCR_014583; [42]), the GRCh37/hg19 genome was aligned with tophat2 [43]. After using samtools (RRID: SCR_002105; [44]) to acquire mapping statistics, quantification of mapped reads and identification of the differentially expressed genes (DEGs) were carried out with cuffdiff 2.1.1 (RRID: SCR_001647; [45]). The DEG table was annotated with gene names from RefSeq Genes (downloaded from UCSC Table Browser, GRCh37/hg19). Gene ontology enrichment analysis was performed with gorilla [46].

### 2.13 Immunofluorescence imaging

The immunofluorescent (IF) staining procedure was conducted in either 12‐well or 24‐well plates. Cells were seeded on the coverslips to obtain 50–70% confluency. Cells were washed twice with PBS and fixed in 4% paraformaldehyde (PFA, Sigma‐Aldrich, cat. #158127) for 15 min at room temperature or with ice‐cold methanol for 10 min at −20 °C. Then, they were washed with ice‐cold PBS three times and incubated with blocking solution: 3% BSA (BioShop Canada, cat. #ALB001.100) in PBS and 0.1% Triton X (Sigma‐Aldrich, cat. #X100‐100ML) for 1 h. After three times PBS wash, samples were first incubated with either anti‐IRF4 (1 : 1000, Cell Signaling, Denvers, MA, USA, Cat. #4964) or anti‐AcTub (1 : 10 000, Sigma‐Aldrich, cat. #T6793) and anti‐ARL13b (1 : 500, Proteintech, Rosemont, IL, USA, cat. #CL488‐17711) antibodies in blocking solution for 1 h at room temperature. After another 3× wash with PBS, samples were incubated with Alexa 488‐ or Alexa 647‐conjugated species‐appropriate secondary antibodies (1 : 1000) together with 4′,6‐diamidino‐2‐phenylindole (DAPI) (1 μg·mL^−1^) diluted in blocking solution for 1 h at room temperature in the dark. After the final PBS wash steps, coverslips are mounted on slides using Mowiol® mounting medium (Sigma‐Aldrich, cat. #81381) or VectaShield® (VectorLabs, Wetzler, Germany, cat. #H‐1000‐10). The samples were visualised on the same day or stored at −20 °C. Image acquisition was done using a Leica confocal microscope (SP8) with a 40× water‐objective lens.

After image acquisition for cilia markers, las x (RRID: SCR_013673 Leica) and image j (NIH, Bethesda, MD, USA) were utilised for quantitative analysis. This was performed by counting DAPI‐stained nuclei representing the number of cells, followed by counting cilia structures based on the AcTub or Arl13b signal from four distinct regions in each sample using the same voltage, gain, and exposure settings. The percentage of cells with cilia was calculated for a total of > 400 DAPI‐marked cells counted for each sample. Image acquisition and analysis were done in a blinded manner.

### 2.14 The Cancer Genome Atlas patient expression and survival data analysis

IRF4 mRNA expression data across pan‐cancer TCGA patient datasets were downloaded through FireBrowse (GDAC Firehose, 2016) and replotted with graphpad prism v8.4 (RRID: SCR_002798).

For survival analysis, overall survival data for patients and matching IRF4 mRNA expression for the top and bottom quartiles of the IRF4 mRNA expression in SKCM TCGA patients were obtained via Xena Browser [47]. The Kaplan–Meier graph and Mantel–Cox statistical analysis were done using graphpad prism (RRID: SCR_002798) software.

To analyse the relation between IRF4 expression DNA methylation signature, IRF4 mRNA expression data for the melanoma cohort [48] was downloaded using Xena Browser. Anti‐log IRF4 expression was calculated, and the patients were grouped based on DNA‐methylation signatures. Mann–Whitney statistical test was applied for statistical analysis between the two groups.

### 2.15 XTT assay

XTT assays (based on the tetrazolium salt sodium 3′‐[1‐(phenylaminocarbonyl)‐3,4‐tetrazolium]‐bis (4‐methoxy6‐nitro) benzene sulphonic acid hydrate) to assess cytotoxicity were used as follows: in a 96‐well plate, 5000 cells were seeded per well. Twelve hours post‐seeding, cells were treated with 5‐Aza, decitabine for 3–5 days, or EPZ‐6438 for 5–7 days. On the day of the assay, the cell medium was exchanged with 100 μL phenol red‐free complete DMEM. XTT assay was carried out with XTT Cell Proliferation Kit II (Roche, cat. #11465015001) according to the manufacturer's protocol.

### 2.16 GFP competition assays

To measure the competitive fitness of the cells upon IRF4 depletion, shRNA transduced cells (coexpressing GFP, i.e., GFP^+^) were mixed with untransduced cells (GFP^−^) at 3 days post‐transduction. Before mixing, the GFP^+^ samples were measured to ensure similar mean fluorescence intensity (MFI) across samples and to determine cell percentage with GFP (%GFP). Upon mixing the GFP^+^ and GFP^−^ cells to obtain 50–60% GFP, the samples were analysed with flow cytometry, %GFP measured, and were assigned as the starting reference point. Mixed cells were reseeded, and every 2–3 days, cells were trypsinised, and an aliquot of them was analysed with flow cytometry to assess %GFP^+^ cells. To calculate the change in GFP^+^ fraction over time, the %GFP^+^ cells at each time point were normalised by the reference point and described as a ratio of 100.

### 2.17 Trypan blue exclusion assays

First, the cell culture media of growing cells were collected into collection tubes to capture detached dying/dead cells. Adherent cells were then washed with 1× PBS; this wash solution was also collected. The remaining adherent cells were detached by trypsinisation. After inactivation with complete DMEM, all cells were collected in the same tube. Cells were pelleted and resuspended in 500–1000 mL PBS. An equal amount of trypan blue (Gibco, Thermo Scientific, cat. #15250061 or Hyclone, Cytiva, cat. #SV30084.01) was mixed with cell suspension, and each sample was measured twice in Countess II FL automated cell counter (Invitrogen) to determine percentages of live and dead cells.

### 2.18 Colony formation assays/clonogenic assays

Three days post‐transduction, 5000 cells were seeded into 6‐well plates for each condition. The culture medium was refreshed once or twice a week, depending on necessity, until single colonies became visible. Cells were fixed with 100% methanol for 20 min on a shaker at room temperature. Cells were washed 1× with ddH_2_O and further stained for 5–10 min on the shaker at RT with 0.5% crystal violet (Sigma‐Aldrich, cat. #HT901‐8FOZ) in 25% methanol. Plates were washed 3× by scooping in excess water and left for drying. Photos were taken of each plate, and crystal violet was further extracted for quantification by incubating plates on the shaker for 20 min at room temperature with 10% acetic acid. The absorbance of extracted crystal violet was measured at 590 nm on the VersaMax microplate reader (Molecular Devices, San Jose, CA, USA).

### 2.19 Real‐time cell analysis

Real‐time cell analysis (RTCA) was performed on an xCELLigence instrument (Agilent). Following transduction, cells were collected, counted, and seeded into RTCA E‐plates. Software and plates were set up according to the manufacturer's protocol, and real‐time proliferation was investigated by detecting a change in surface impedance proportional to cell number. Impedance measurements were automatically taken every 30 min for several days until wells became confluent (the plateau stage of the growth curve). At the end of the experiment, cell index values were normalised to the starting point of the experiment. Data from multiples of 6 h were transferred to graphpad and visualised.

### 2.20 Cell cycle analysis

Cells were seeded in 6 cm plates. On the day of the assay, cells were trypsinised and counted with Countess II FL (Invitrogen). 1–1.5 × 10^6^ cells were collected for each sample. Cells were then centrifuged and washed with ice‐cold PBS twice. After the final wash and centrifugation cycle, the supernatant was discarded, and 750 μL of freshly prepared ice‐cold 70% Ethanol was added to the samples while slowly vortexing. Samples were stored at −20 °C until the day of flow cytometry analysis. Before flow cytometry analysis with FACS Calibur (BD), samples were centrifuged and washed with ice‐cold PBS twice again. After the final wash, cell pellets were resuspended in 500 μL of filtered PBS with freshly added propidium iodide (50 μg·mL^−1^, Invitrogen) and RNase A (100 μg·mL^−1^, Thermo). Samples were incubated at 37 °C for 1–2.5 h, depending on the cell line. Samples were then analysed with FACS Calibur (BD). Cell cycle phases were analysed using flowjo 10.8 (FlowJo LLC, Ashland, OR, USA, RRID: SCR_008520) software. Univariate modelling‐Watson Pragmatic algorithm was used to determine cell cycle phases.

### 2.21 Statistical analyses

Statistical analysis approaches are described for each assay, wherever relevant, in the corresponding section or figure legend. n.s., not significant; *P < 0.05; **P < 0.01; ***P < 0.001; ****P < 0.0001.

## 3 Results

### 3.1 IRF4 expression is common in melanoma and is associated with dependency and poor patient survival

**Fig. 1:**
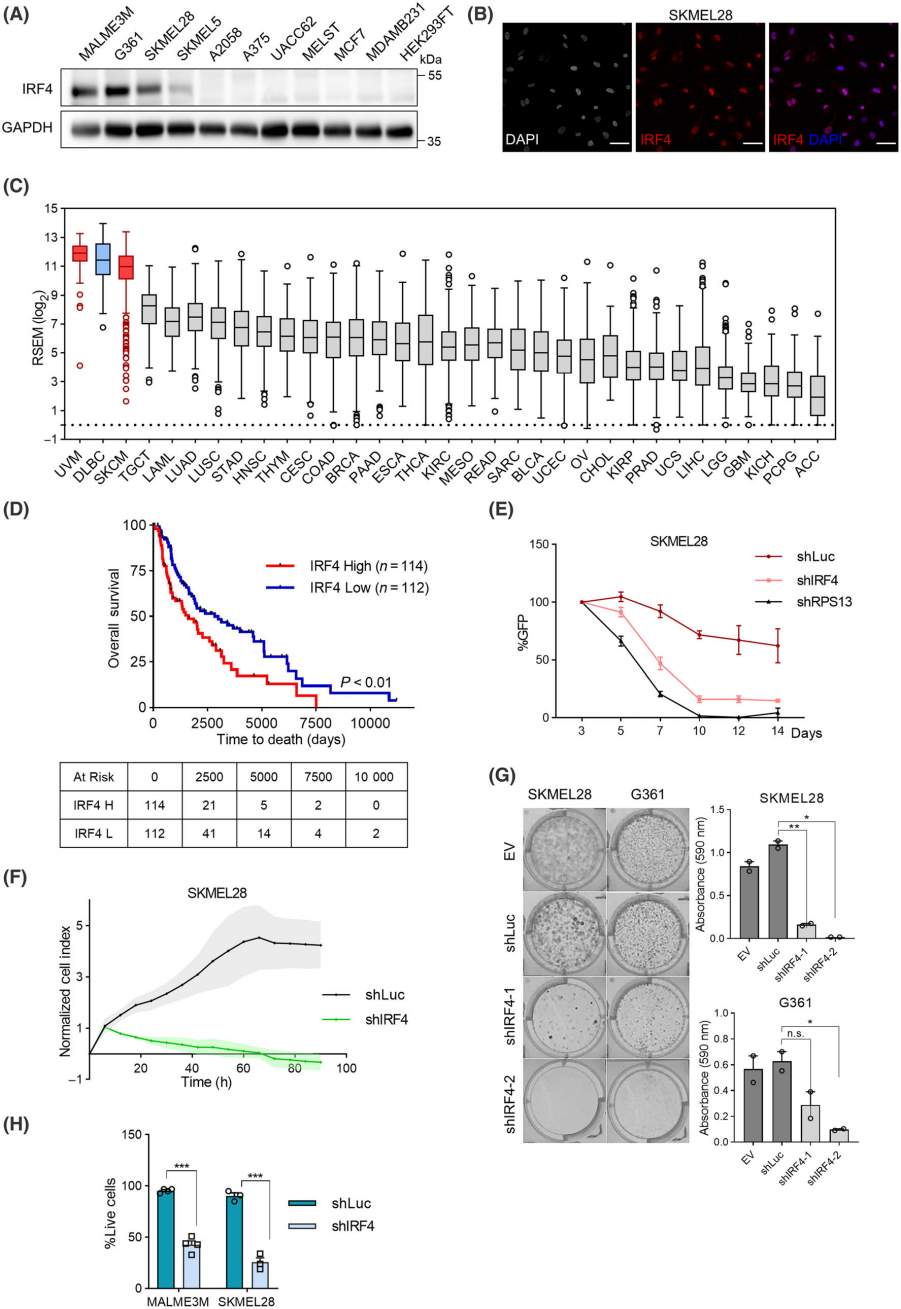
IRF4 expression is common in melanoma and is associated with dependency and poor patient survival. (A) IRF4 protein levels in a panel of melanoma (MALME3M, G361, SKMEL28, SKMEL5, A2058, A375, and UACC62), melanocyte (MELST), and non‐melanoma cell lines (MCF‐7, MDAMB231, HEK293FT) investigated by immunoblotting. Representative data from three independent experiments are shown. (B) Sub‐cellular localization of IRF4 as shown by immunofluorescence (IF) staining of SKMEL28 melanoma cells with Alexa 647 conjugated anti‐IRF4 antibody and DAPI, coloured in red and grey/blue, respectively. Scalebar: 50 μm. Representative confocal immunofluorescence microscopy images from three independent experiments. (C) Analysis of IRF4 mRNA levels from RNA‐seq data of TCGA patient cohorts. DLBCL, diffuse large B‐cell lymphoma; SKCM, skin cutaneous melanoma; UVM, uveal melanoma. For other abbreviations, refer to https://gdc.cancer.gov/resources‐tcga‐users/tcga‐code‐tables/tcga‐study‐abbreviations. (D) Kaplan–Meier curve of overall survival for IRF4^high^ (red) and IRF4^low^ (blue) TCGA melanoma patients. The number of patients in each group is indicated inside parentheses, corresponding to the top (red) and bottom (blue) quartiles of IRF4 expression. (E) GFP competition assay for the assessment of competitive fitness of SKMEL28 IRF4 depleted cells *versus* control cells. The proportion of cells stably co‐expressing GFP with an RNAi trigger against IRF4 (shIRF4) to unmanipulated cells is monitored over time to gauge relative cell fitness. Cells with an RNAi trigger against the firefly luciferase gene (shLuc) are used as a negative control, whereas cells with an RNAi trigger against an essential ribosomal component (shRPS13) are used as the positive control. The average values of five independent experiments (normalised to day 3) are plotted. (F) Real‐time cell analysis with the XCELLigence® instrument to gauge the effect of cell accumulation in IRF4‐depleted melanoma cells. Error bars shown as area fill. (G) Colony formation assays in IRF4‐depleted samples. Left: representative crystal violet‐stained colonies, right: quantification of extracted crystal violet signal. (H) Trypan blue exclusion assays to assess dead cell fraction with IRF4 knockdown. Data Information: In (C), whiskers are plotted using the Tukey method. In (D), the *P*‐value of 0.0013 was calculated by the log‐rank (Mantel–Cox) test. In (E), two‐way ANOVA with multiple comparisons using the Dunnet method. The error bards depict SEM of five independent experiments. The adjusted *P*‐values for shIRF4 vs. shLuc are each < 10^−4^ for days 7, 10, 12, 14, and for day 5, *P* = 0.023. In (F), error bars depict SEM of 4 independent experiments. In (G), error bars are SEM from 2 independent experiments. In (H), MALME3M and SKMEL28 data are from four and three independent experiments, respectively. Error bars are shown as SEM. In (G, H), Welch's *t*‐test was performed. In (G), *P*‐values for shIRF4‐1 and shIRF4‐2 compared to shLuc are 0.0086 and 0.013, respectively, in SKMEL28, and 0.064 and 0.043 in G361 cells, respectively. In (H), *P*‐values for MALME3M and SKMEL28 are 0.0002 and 0.0003, respectively.

Encouraged by the early literature showing IRF4 expression in melanoma [23, 24, 25] we screened a panel of cell lines, consisting mostly of cutaneous melanoma lines, for IRF4 protein expression by immunoblotting (Fig. 1A). Here, we observed IRF4 expression in most, but not all, of the melanoma cell lines tested, while no IRF4 expression was observed in breast cancer, immortalised melanocyte and kidney cell lines. Nuclear localisation is a prerequisite for transcription factor activity. Previous work demonstrated mainly nuclear localisation of IRF4 in the histological samples of melanoma patients [25]. To assess the subcellular localisation of IRF4 in melanoma cell lines, we performed immunofluorescence (Fig. 1B; Fig. S1A) and immunoblotting after subcellular fractionation (Fig. S1B), where we observed essentially nuclear localisation of IRF4. We also investigated IRF4 mRNA levels across cancer types in patient tumour samples in The Cancer Genome Atlas (TCGA; [49]) datasets. Melanoma tumours displayed the highest average IRF4 expression along with lymphocyte‐derived cancers (Fig. 1C), consistent with similar analyses on the Cancer Cell Line Encyclopedia (CCLE) [50] dataset by us (Fig. S1C) and others [11]. Furthermore, we observed that melanoma patients with high IRF4 expression in tumours had worse overall survival in the TCGA skin melanoma dataset (Fig. 1D) and in another published melanoma dataset [51] (Fig. S1D).

Since we initially identified highly expressed IRF4 as a dependency factor in immune cell derived cancers through loss‐of‐function drop‐out screens [5, 6], we examined comparable datasets for IRF4 in melanoma. Accordingly, we identified IRF4 as a dependency factor in melanoma cell lines (along with lymphocyte‐derived cancer lines) in a database of integrated CRISPR/Cas9 cancer dependency screens (DepMap; [52]). Here, we observed that IRF4 levels significantly correlated with higher dependency (i.e., lower gene effect/Chronos scores; Fig. S1E), suggesting a melanoma cell‐intrinsic dependency on IRF4 for IRF4‐expressing melanomas, similar to what was previously observed for lymphocyte‐origin cancers. We verified this dependence of IRF4‐expressing melanoma cell lines on IRF4 expression by monitoring the ratio of IRF4‐depleted cells to control cells (i.e., via GFP competition assay; Fig. 1E; Fig. S1F). To verify the dependency role of IRF4 via orthogonal approaches, we asked whether experimental manipulation of IRF4 levels would impact proliferation or cell survival *in vitro*. Accordingly, IRF4 depletion negatively impacted cell accumulation in real‐time cell analysis (Fig. 1F; Fig. S1G) and colony forming ability (Fig. 1G), and increased cell death in trypan dye exclusion assays (Fig. 1H; Fig. S1H), consistent with CRISPR screen data and GFP competition analyses.

Taken together, these observations suggest that not only IRF4 is highly expressed in a substantial proportion of melanomas but also it acts as a dependency factor in these cells.

### 3.2 IRF4 modulates DNA and histone H3 lysine 27 methylations in melanoma cells

To gain insight into the pathways and processes by which IRF4 exerts its cancer‐critical functions in melanoma cells, we performed transcriptome sequencing (RNA‐seq) with SKMEL5 and SKMEL28 melanoma cell lines after short‐term (i.e., 3 days) shRNA‐mediated IRF4 knockdown. Genes regulated by IRF4 in these cell lines showed considerable overlap (Tables S4 and S5). To flesh out common enriched processes, we used a method for the analysis of gene ontology (GO) category enrichments in the differentially expressed gene (DEG) lists that aims to highlight the relations among GO categories [46] for the sets of DEGs shared by both cell line data (Fig. S2A,B; Tables S6 and S7). For instance, among the IRF4‐repressed DEGs (i.e., genes whose expression increase upon IRF4 depletion), the two cell lines shared enrichment for sterol/lipid metabolic pathways (Fig. S2B). IRF4's roles in these pathways were previously addressed both in physiological [53] and cancer [5] contexts. On the other hand, common IRF4‐activated DEGs were enriched for several GO categories, notably for those related to mitotic cell division cycle (Fig. S2A), in keeping with IRF4's cancer dependency status. Another category enriched for IRF4‐activated DEGs in both melanoma cell lines, namely covalent chromatin modification and related categories (Fig. S2A), have not previously been described in the context of IRF4 to our knowledge, and therefore piqued our interest for further investigation.

**Fig. 2:**
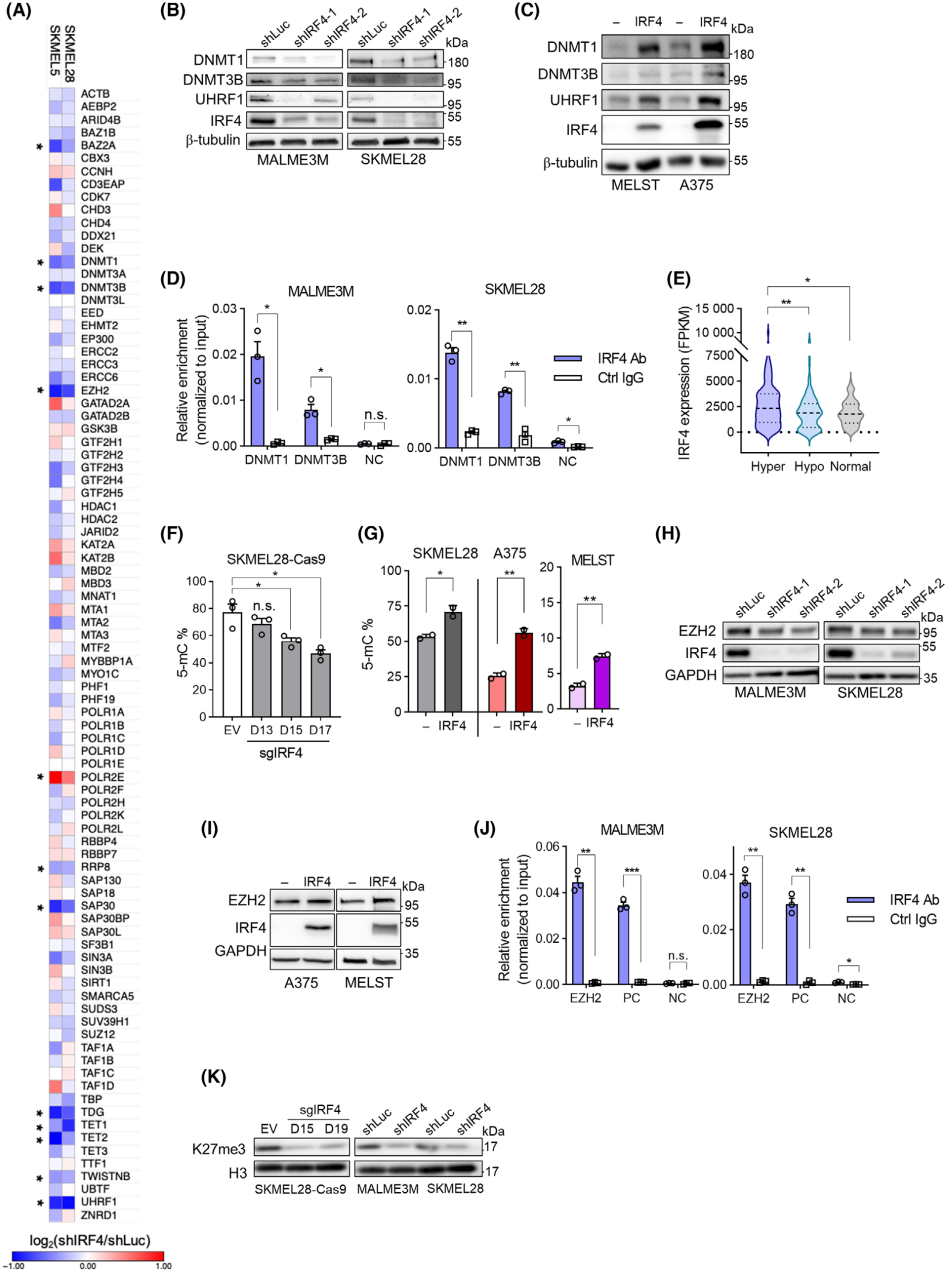
IRF4 modulates DNA and histone H3 Lysine 27 methylations in melanoma cells. (A) Heatmap representation of RNA‐seq results of genes categorised as “Epigenetic Regulation of Gene Expression” (R‐HSA‐212165; except genes encoding histones) in the Reactome database [54], showing expression alterations of chromatin factors upon IRF4 knockdown. “*” denotes genes whose expression significantly altered (FDR *q* < 0.001) in both cell lines. (B) Western blot analysis of DNMT1, DNMT3B, and UHRF1 expression upon IRF4 depletion with two different lentivirally delivered shIRF4s, or shLuc as a non‐targeting control. (C) Western blot analysis of DNMT1, DNMT3B, UHRF1 expression in lentivirally delivered doxycycline‐inducible IRF4 overexpressing cell lines. ‘–’, control cells; ‘IRF4’, IRF4 overexpressing cells. (D) ChIP‐qPCR analysis with anti‐IRF4 antibody at *DNMT1* promoter and *DNMT3B* intronic loci. Negative control (NC) refers to an intergenic region with no evidence of IRF4 binding. Ctrl IgG refers to control ChIPs with isotype‐matched normal (unimmunized) IgG antibody. (E) TCGA melanoma data for IRF4 expression in patient tumours pre‐classified as hypermethylated (hyper) DNA signature compared to those pre‐classified as hypomethylated (hypo) and normal DNA methylation. (F) Genomic DNA 5‐cytosine methylation percentages via ELISA with 5‐mC antibody on genomic DNA (gDNA) extracted from CRISPR/Cas9‐based IRF4 knockout SKMEL28 cells. EV, empty vector (white), and sgIRF4 cells transduced with a guide RNA for *IRF4* locus (grey). D, days after transduction. (G) 5‐mC ELISA on gDNA samples obtained from IRF4‐overexpressing cell lines. (H) Western blot analysis of EZH2 protein levels in IRF4 knockdown cell lines. (I) Western blot analysis of EZH2 protein levels in lentivirally delivered doxycycline‐inducible IRF4 overexpressing cell lines. (J) ChIP‐qPCR analysis with anti‐IRF4 antibody at *EZH2* locus. Positive control (PC) is the tyrosinase (Tyr) promoter [15]. Negative control (NC) is from an intergenic region with no observable IRF4 binding. Representative data from three independent experiments are shown. (K) Western blot analysis of histone H3 lysine 27 tri‐methylation (H3K27me3) levels with IRF4 knockout or knockdown cells. ‘D15’ and ‘D19’ refer to days 15 and 19, respectively, following doxycycline‐induced Cas9 expression for knocking out IRF4 with a cognate single guide RNA (sgIRF4). Total histone H3 is loading control. In (B, C, H, I, K), representative western blots are shown from three independent experiments. Data information: In (D), error bars depict SEM of three independent biological replicates. Statistical analysis with Welch's *t*‐test (i.e., unpaired Student's *t*‐test for unequal variance). In the MALME3M plot, the *P*‐values for DNMT1, DNMT3B, and NC are 0.013, 0.013, and 0.3848, respectively. In the SKMEL28 plot, the *P*‐values for DNMT1, DNMT3B, and NC are 0.0011, 0.0018, and 0.0105, respectively. In (I), ** Hyper vs. hypo *P* = 0.0091, * Hyper vs. normal *P* = 0.019 by Mann–Whitney test. In (F), Error bars depict SEM from three independent experiments. Welch's *t*‐test between sgIRF4 vs. EV samples. The *P*‐values for D13, D15, and D17 are 0.16, 0.030, and 0.012, respectively. In (G), Error bars are SEM from two independent experiments and *P*‐values by Welch's *t*‐test for SKMEL28, A375, and MELST are 0.039, 0.0047, and 0.0049, respectively. In (J), error bars depict the SEM of three independent biological replicates. Statistical analysis with Welch's *t*‐test. In the MALME3M plot, *P*‐values of 0.0016, 0.0008, and 0.38 correspond to EZH2, PC and NC. In the SKMEL28 plot, *P*‐values for the same three loci are 0.0026, 0.0018, and 0.011, respectively.

Consistent with the high‐level GO enrichment analysis described above, expression of several genes involved in chromatin modifications, and hence in epigenetic gene regulation [54], was modulated by IRF4 knockdown in either one or both of the melanoma cell lines used in the transcriptomic analysis (Fig. 2A; Table S8). Among these, decreases in the expression of genes encoding cellular machinery central to DNA methylation, namely *DNMT1*, *DNMT3B* and *UHRF1*, were observed in both cell lines. *DNMT1* and *DNMT3B* encode two of the three functional DNA methyltransferase enzymes in the human genome, primarily responsible for maintenance and *de novo* DNA methylation, respectively [55], whereas UHFR1 is an essential cofactor for DNMT1 [56]. 5‐Cytosine methylation of DNA is the widely studied epigenetic modification in cancers, and melanoma is no exception [57, 58, 59]. Encouraged by these observations, we set out to directly test the role of IRF4 in DNA methylation. We first confirmed the effects of experimental manipulation of IRF4 expression on DNMT1, DNMT3B, and UHRF1 levels at mRNA (Fig. S2C) and protein levels (Fig. 2B,C; Fig. S2D–G) in an expanded set of cell lines with high IRF4 expression (i.e., SKMEL28, MALME3M, G361) and those with low/undetectable IRF4 expression (i.e., the MITF expression negative A375, the immortalised melanocyte line MELST, and UACC62) with lentivirally delivered shRNA for knockdown or doxycycline‐induced ectopic IRF4 overexpression. We hypothesised that IRF4 regulation of the DNMT genes might be direct, since publicly available ENCODE data [60] suggested that both loci contained accessible chromatin regions in the melanocytic lineage that coincided with predicted IRF binding motifs. Using PCR primers spanning these candidate regions, we observed IRF4 localisation to both *DNMT1* and *DNMT3B* proximal loci via chromatin immunoprecipitation (ChIP) (Fig. 2D). In addition, we identified data supporting a role for IRF4 in DNA methylation: TCGA melanoma patient tumours classified as “hyper‐methylated” displayed overall higher IRF4 expression than those classified as “hypo‐methylated” or “normal” (Fig. 2E). Accordingly, we directly examined global DNA methylation levels in melanoma cell lines using a corresponding enzyme‐linked immunosorbent assay (ELISA). Experimental manipulation of IRF4 expression altered global methylation levels in a manner consistent with its effects on the expression of DNA methylation genes (Fig. 2F,G).

The histone H3 lysine 27 methyltransferase EZH2 is among the common IRF4 transcriptional target chromatin modifiers that we identified through RNA‐seq analysis (Fig. 2A; Tables S4 and S8). Di‐ and tri‐methylation of histone H3 lysine 27 (H3K27me) are gene‐repressive modifications frequently dysregulated in cancers that can contribute to malignancy, mostly in an oncogenic capacity [61]. This is predominantly due to the overactivity of the polycomb repressive complex 2 (PRC2) catalytic subunit EZH2, either due to specific gain‐of‐function point mutations or, more commonly, due to overexpression [62]. EZH2 overactivity is also implicated in melanoma progression and invasiveness, mainly through repressive activity on diverse downstream targets, including those implicated in cell differentiation, immune regulation, and cell cycle control [63, 64]. Through experimental manipulation of IRF4 expression, we confirmed the regulation of EZH2 expression by IRF4 in various melanoma and melanocytic cell lines at mRNA (Fig. S2C) and protein levels (Fig. 2H,I; Fig. S2D–G). To gain insight into whether IRF4 regulation of EZH2 expression is direct, we screened published ENCODE chromatin accessibility data in melanocytic cells for candidate regions of predicted IRF binding and designed primer pairs spanning candidate regions for ChIP‐qPCR. Among these, one of the intragenic candidate regions at the *EZH2* locus showed reproducible and significant enrichment for IRF4 binding in ChIP‐qPCR assays (Fig. 2J), consistent with a direct regulation of EZH2 expression by IRF4. Since EZH2 is the major enzyme for the repressive H3K27me, we asked whether IRF4 regulation of EZH2 leads to changes in the cognate H3K27me levels. Indeed, we observed by immunoblotting that experimental down‐modulation of IRF4 expression leads to a decrease in the bulk levels of H3K27 trimethylation (Fig. 2K), with no discernible changes in the level of unrelated histone modification, H3K4me1 (Fig. S2G). While increased bulk H3K27me3 levels via immunoblotting upon IRF4 overexpression could only be observed in MELST (a cell line with very low basal H3K27me3, unpublished observations), substantial increases in H3K27me3 were detected in a locus specific manner with ChIP‐qPCR in both MELST and A375 (see below).

Collectively, these findings point to IRF4's contribution to extensive gene silencing by enabling repressive chromatin in melanoma cells through activation of the expression of key epigenetic factors responsible for DNA and histone methylations.

### 3.3 IRF4 regulates multiple melanoma‐critical tumour suppressor genes and the cell cycle

**Fig. 3:**
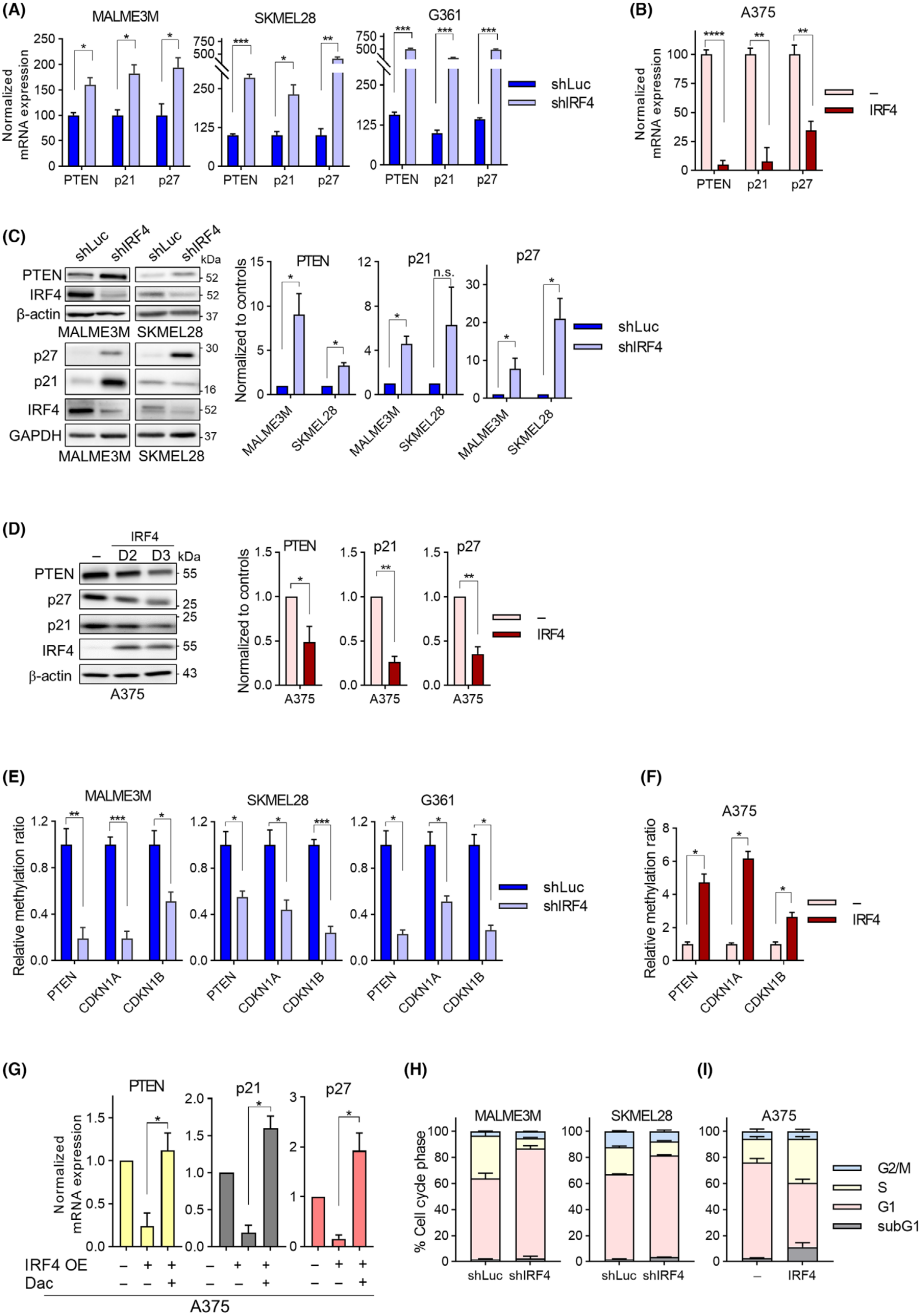
IRF4 regulates multiple melanoma‐critical tumour suppressor genes and the cell cycle. (A) RT‐qPCR analysis of PTEN, p21, and p27 expression at day 6 of IRF4 knockdown cells. (B) RT‐qPCR analysis of PTEN, p21, and p27 expression in IRF4 overexpressing cells. (C) Western blot analysis at day 6 of IRF4 knockdown for the expression of p21 and p27, and PTEN. Quantification of western blot from three different experiments are on the right side. (D) Western blot analysis at day 2 and day 3 of IRF4 overexpression for PTEN, p27, and p21 expression. Representative data from three independent experiments are shown. Quantification of western blot from three different experiments is on the right side. (E, F) MSRE‐qPCR analysis of promoter methylation levels at *PTEN*, *CDKN1A* (encoding p21), and *CDKN1B* (encoding p27) in (E) IRF4 depleted and (F) IRF4 overexpressing cells. (G) RT‐qPCR analysis of PTEN, p21 and p27 genes in A375 cells. After 2 days of doxycycline‐induced IRF4 overexpression, cells were treated with decitabine (Dac; 500 ng·mL^−1^) or vehicle control (DMSO) together with doxycycline for another 3 days. (H, I) Propidium Iodide (PI) stained and fixed cells analysed by flow cytometry for DNA content to assess cell cycle phase profiles of (H) IRF4 depleted and (I) IRF4 overexpressing cells. Data Information: In (A–G), statistical analysis with Welch's *t*‐test. In (A, B), error bars depict SEM from three independent experiments. For PTEN, p21, and p27, *P*‐values are 0.0185, 0.0113, 0.0179 in MALME3M; 0.0004, 0.0199, 0.0013 in SKMEL28; < 10^−4^, 0.0037, 0.0024 in A375, respectively. In (C, D), error bars depict SEM from three independent experiments. (C), *P*‐values for PTEN, p21, and p27 are 0.038, 0.046 and 0.043 in MALME3M; and 0.0110, 0.1814, and 0.033 in SKMEL28. In (D), *P*‐values in A375 for PTEN, p21 and p27 are 0.032, 0.004, and 0.008 respectively. Error bars depict SEM from three (E, I) and two (F–H) independent experiments. In (E, F), *P*‐values for PTEN, CDKN1A, and CDKN1B are 0.0054, 0.0004, 0.016 in MALME3M; 0.022, 0.014, 0.0003 in SKMEL28; 0.039, 0.049, 0.022 in G361; and 0.035, 0.023, 0.033 in A375, respectively. In (G) *P*‐values for PTEN, p21 and p27 are 0.0412, 0.0141, and 0.0481 respectively. In (H, I), two‐way ANOVA with Bonferroni's multiple comparison tests with *P* < 10^−4^ for changes in cell cycle phases between samples in each plot.

We sought to understand the downstream consequences of IRF4‐mediated epigenetic silencing in melanoma cells. Since promoter methylation‐mediated silencing of tumour suppressor genes (TSGs) is a widely observed phenomenon that can play a causal role in cancer [65], including melanoma [58, 66, 67], we first investigated whether IRF4 was involved in this process. To this end, we experimentally manipulated IRF4 expression in melanoma cells at time scales where downstream effects of altered repressive methylations on gene expression are expected to manifest (i.e., ~ 1 week for IRF4 knockdowns and several days for overexpressions), so that cells had sufficient time to for the effects of IRF4 expression manipulations to reveal not only on IRF4 levels but also on DNMT, UHRF1 levels, and on the average at least one cell cycle has been transversed for the methylation changes to occur. We screened by RT‐qPCR a panel of well‐studied TSGs whose expression is known to be repressed by promoter DNA hypermethylation in melanoma. Among the TSGs tested, the expression of the cell cycle regulatory cyclin‐dependent kinase inhibitors (CKIs) CDKN1A (p21/Cip1/Waf1), CDKN1B (p27/Kip1), and the phosphoinositide 3‐kinase (PI3K) pathway negative regulator lipid phosphatase PTEN were altered by manipulation of IRF4 expression, such that IRF4 depletion generally led to increases in the expression of TSGs, whereas IRF4 overexpression led to decreases, both at mRNA and protein levels (Fig. 3A–E; Fig. S3A–D). Expression of these genes were also responsive to DNA methyltransferase inhibitor (DNMTi) treatment (Fig. S3E), further supporting the role of DNA methylation in their control. Using TCGA SKCM methylation data, we identified candidate CpG islands for DNA hypermethylation in the promoter regions of these TSGs. We used methylation sensitive restriction enzyme analysis (MSRE‐qPCR) to analyse promoter methylation at these TSG CpG islands. We observed altered promoter methylation following manipulation of IRF4 expression, in line with IRF4's role in modulating DNA methylation via DNMT1/3B and UHRF1 expression. For instance, IRF4 knockdown led to a decrease in promoter methylation (i.e., drop in MSRE‐qPCR signal), and the opposite was observed with IRF4 overexpression (Fig. 3E,F; Fig. S3F). Equivalent input DNA use was ensured with the analysis of undigested genomic DNA (Fig. S3G,H). To verify that IRF4‐dependent regulation of the expression of TSGs involve DNA methylation, we performed a complementation experiment where IRF4 overexpression was combined with DNMT inhibitor treatment. Here, we observed that the repression on the expression of TSGs that IRF4 overexpression caused was reversed when combined with the DNMTi decitabine (Fig. 3G; Fig. S3I), providing further evidence on the role of DNA methylation in IRF4‐mediated TSG repression.

The influence of IRF4 on promoter methylation and expression of CKI TSGs led us to investigate the potential impact of IRF4 on cell cycle regulation in melanoma cells. For this, we analysed DNA content by propidium iodide staining and flow cytometry analysis to gauge the cell cycle phase profiles of cells with experimentally manipulated IRF4 expression. We observed an increase in the proportion of cells in the G1 phase of the cell cycle with a concomitant decrease in S‐phase in IRF4‐depleted cells (Fig. 3H), consistent with cell cycle arrest or progression delay. The opposite pattern was evident upon IRF4 overexpression (Fig. 3I; Fig. S3J).

### 3.4 IRF4 is an upstream regulator of WNT/β‐catenin pathway in melanoma cells

**Fig. 4:**
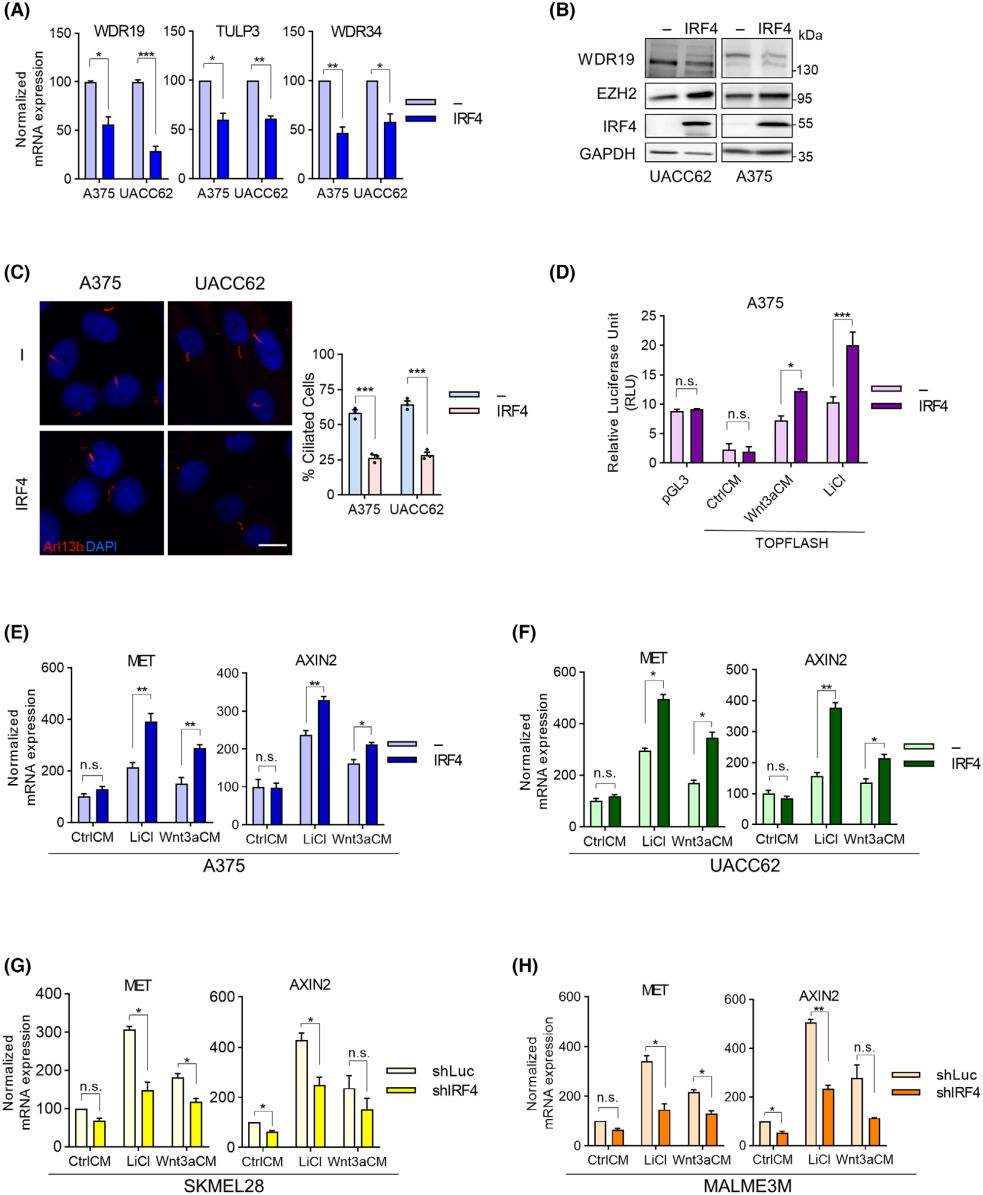
IRF4 is an upstream regulator of WNT/β‐catenin pathway in melanoma cells. (A) RT‐qPCR and (B) Western blot analysis of WDR19 expression upon IRF4 overexpression. Repesentative western blot from three independent experiments. (C) Effect of IRF4 overexpression on ciliation. Representative confocal immunofluorescence microscopy images of 48‐h serum‐starved cells. Percentage of ciliated cells defined by staining for Arl13b (red) and DAPI. Scale bar: 5 μm. Left: representative images, right: ciliated cell quantification from three (A375) and two (UACC62) independent experiments ± SEM (for each experiment, > 400 cells counted). (D) Luciferase reporter assays for the assessment of β‐Catenin transcriptional activity using the TOPFLASH plasmid (with 3× TCF/LEF binding sites) in A375 cells upon IRF4 overexpression, combined either with WNT3a‐conditioned medium (WNT3aCM) or with 20 mm LiCl treatment. pGL3 is a β‐Catenin‐independent constitutively active control plasmid. (E–H) RT‐qPCR analyses for two β‐Catenin target genes, AXIN2 and MET, with IRF4 overexpression in (E) A375 and (F) UACC62, and with IRF4 knockdown in (G) SKMEL28 and (H) MALME3M cells. Data Information: In (A, C, E–H), statistical analysis with Welch's *t*‐test. In (D), two‐way ANOVA with Bonferroni's multiple comparisons. In (A), error bars depict SEM from three independent experiments and *P*‐values for WDR19, TULP3 and WDR34 are 0.0157, 0.012, and 0.006 for A375; and 0.0008, 0.002, 0.019 for UACC62. In (C), error bars depict SEM from three independent experiments. In A375 and UACC62, the *P*‐value is 0.0002 for both sets. In (D), error bars depict SEM from two independent experiments. The *P*‐values for Wnt3aCM, and LiCl are 0.034, and 0.0006 respectively. In (E–H), error bars depict SEM of three independent experiments for A375 and two independent experiments for UACC62, SKMEL28 and MALME3M. In (E), *P*‐values for CtrlCM, LiCl, and Wnt3aCM are 0.073, 0.0061, 0.0060 in the MET plot; 0.93, 0.0016, 0.012 in the AXIN2 plot, respectively. In (F), *P*‐values for CtrlCM, LiCl, and Wnt3aCM are 0.311, 0.011, 0.015 in the MET plot and 0.18, 0.0073, 0.020 in the AXIN2 plot, respectively. In (G), *P*‐values for CtrlCM, LiCl, and Wnt3aCM are 0.061, 0.0256, 0.0201 in the MET plot and 0.04, 0.0261, 0.1671 in the AXIN2 plot, respectively. In (H), *P*‐values for CtrlCM, LiCl, and Wnt3aCM are 0.062, 0.0133, 0.0134 in the MET plot and 0.044, 0.0029, 0.9841 in the AXIN2 plot, respectively.

We also wanted to understand how IRF4 regulation of EZH2 expression and H3K27me levels might contribute to melanoma. EZH2 is known to act in an oncogenic capacity through multiple pathways in melanoma [63, 64]. With a candidate approach, we focused on a recently identified pathway in melanoma in which EZH2 contributes to oncogenic WNT/β‐catenin signalling [68, 69] via epigenetic repression of primary cilia formation [70], a negative regulator of β‐catenin signalling. In the context of primary cilia, the *WDR19/IFT144* gene encodes a component of the intraflagellar transport machinery [71], is a candidate TSG and a top EZH2 repression target in melanoma [70, 72]. Additional genes encoding primary cilia components that were reported to be EZH2 dependent include WDR34 and TULP3 [70]. We verified EZH2's role on the expression of these genes with EZH2 overexpression (Fig. S4A). We then focused on *WDR19* as an EZH2 target cilium gene, and tested in the IRF4‐expressing SKMEL28 and MALME3M melanoma cell lines with ChIP‐qPCR H3K27me3 levels at *WDR19* promoter (Fig. S4B), which decreased with IRF4 depletion (Fig. S4C), and increased with ectopic IRF4 overexpression in IRF4 nonexpressing cells (Fig. S4D). We then investigated the effect of experimental manipulation of IRF4 expression on these primary cilium component genes (Fig. S4E–G; Fig. 4A,B), obtaining results consistent with an EZH2‐driven repressive effect of IRF4 on the expression of genes encoding cilium components. We further tested EZH2 dependence on IRF4's role on the expression of these genes by combining EZH2 overexpression with IRF4 knockdown (Fig. S4H). The results of these experiments support at least partial contribution of EZH2 in the IRF4‐modulated regulation of the expression of genes encoding primary cilium components.

We then asked if the influence of IRF4 on primary cilium components' expression extends to primary ciliogenesis and β‐catenin signalling. For ciliogenesis, we could only study the IRF4‐nonexpressing lines, since the IRF4‐expressing melanoma lines that we used in this study lacked observable primary cilia to begin with [70] and could not be sustained under the experimental conditions combined with IRF4 depletion (unpublished observations). First, we observed primary cilia by IF colocalisation of cilia components acetylated tubulin (AcTub) and ARL13B (Fig. S4I), and validated the previously noted positive effect of pharmacological EZH2 inhibition (EZH2i) on primary cilia formation [70] by anti‐ARL13B staining (Fig. S4J). We then examined the effect of experimental IRF4 overexpression on primary cilia formation, observing a decreased proportion of cells displaying primary cilia (Fig. 4C) and a partial reversal of the effects of EZH2i upon IRF4 overexpression (Fig. S4J–L), in line with the role of IRF4 in the regulation of EZH2 expression and H3K27 methylation. This raised the possibility that IRF4 could modulate the WNT/β‐catenin pathway in melanoma cells due to the previously described [70] primary cilium regulation of WNT/β‐catenin pathway. To test this, we used the TOPFLASH reporter assay where the firefly luciferase gene is under the control of TCF/LEF binding sites, to gauge β‐catenin pathway transcriptional activity [73]. Well‐established activators of this pathway, namely the GSK‐3β inhibitor LiCl [74] and a cell culture supernatant containing the pathway ligand WNT3A (produced by heterologous expression in L929 cells) were able to increase the luciferase signal in melanoma cells (Fig. 4D). More importantly, overexpression of IRF4 further increased the luciferase signal (Fig. 4D) while not showing any effect on the transcriptional output of the constitutively active control pGL3 construct. IRF4's effect was also evident at endogenous β‐catenin target genes in melanoma [75], namely *AXIN2* and *MET*, with both IRF4 overexpression (Fig. 4E,F) and IRF4 depletion (Fig. 4G,H), consistent with a positive regulatory role of IRF4 on the oncogenic WNT/β‐catenin pathway at least partly through the EZH2‐H3K27me‐primary cilium route.

### 3.5 IRF4 is an upstream regulator of AKT pathway in melanoma cells

**Fig. 5:**
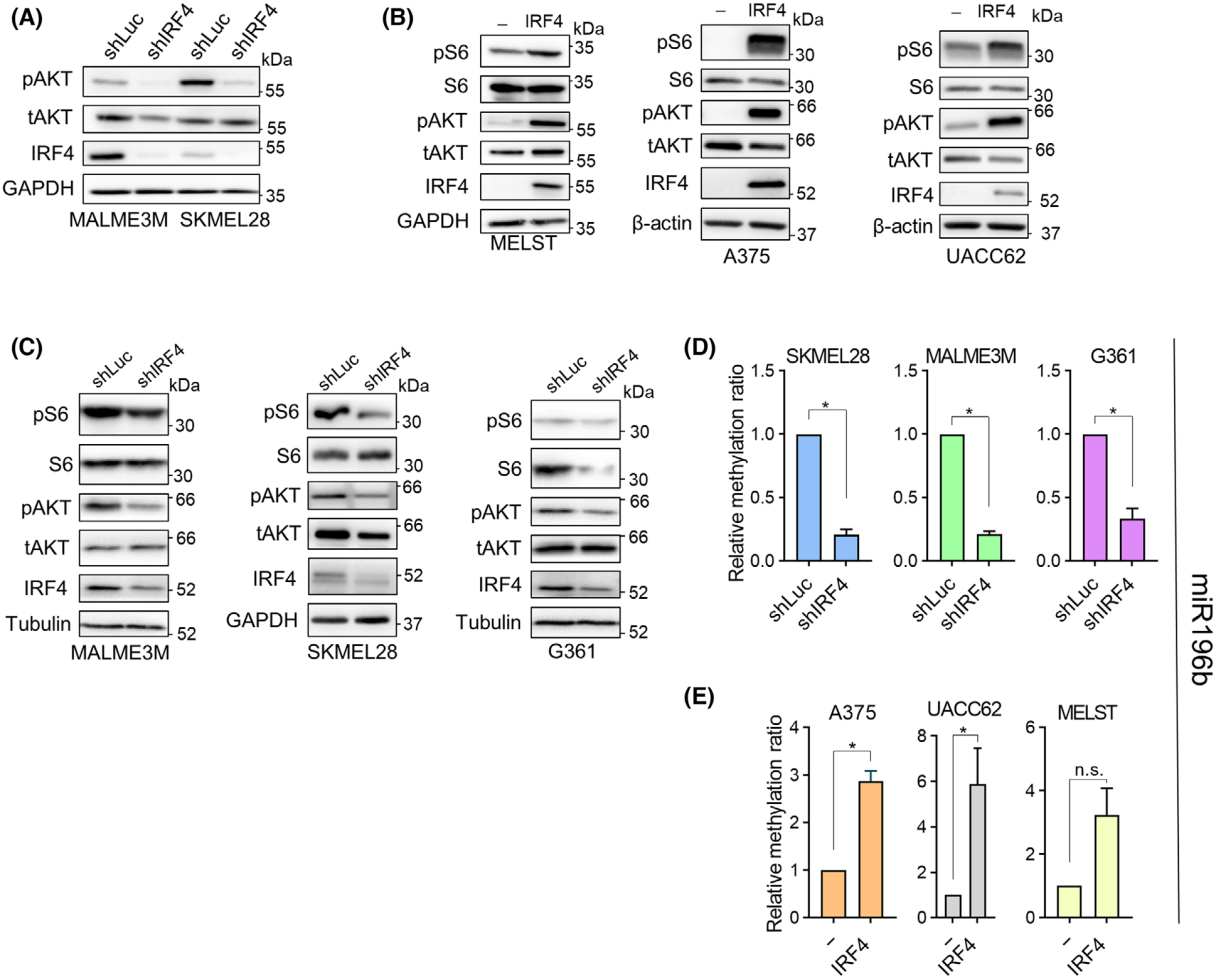
IRF4 is an upstream regulator of AKT pathway in melanoma cells. (A) Western blot analysis of Ser473‐phosphorylated AKT (pAKT) levels upon IRF4 knockdown. Total AKT (tAKT) as an additional loading control. (B) Western blot analysis of IRF4 overexpressing cells for pAKT and Ser235/236‐phosphorylated S6 protein (pS6) levels. (C) Western blot analysis of IRF4 knockdown cells for pAKT and pS6. (A–C: Representative blots from two to three independent experiments). (D, E) MSRE‐qPCR for miR196b promoter in (D) IRF4 knockdown and (E) IRF4 overexpressing cells. Data Information: In (D, E), statistical analysis with Welch's *t*‐test. In (D, E), error bars depict SEM from two independent experiments. In (D) *P*‐values are 0.0179, 0.0101, 0.0395 in SKMEL28, MALME3M and G361 respectively. In (E) *P*‐values are 0.0256, 0.0483, 0.0833 in A375, UACC62 and MELST respectively.

The observation of PTEN as an indirect repression target of IRF4 in melanoma cells (e.g., Fig. 3A–E) prompted us to investigate the effect of IRF4 on the AKT (Protein Kinase B) pathway, where PTEN acts as an upstream negative regulator by negating the effects of the positive regulator phosphoinositide 3‐kinases (PI3Ks). The PI3K‐AKT pathway is yet another oncogenic pathway implicated in melanoma, for instance through its positive role on cell growth, proliferation and survival [76]. Therefore, we first investigated whether experimental manipulation of IRF4 expression modulated some of AKT pathway's key molecular activity markers. Phosphorylation of AKT is a necessary step for the activation of its kinase function [77]. Consistent with the repressive influence of IRF4 on the expression of the pathway's negative regulator PTEN, we observed decreased phospho‐AKT levels upon IRF4 knockdown, and elevated levels upon overexpression (Fig. 5A,B). A major branch downstream of the AKT pathway culminates in the phosphorylation and activation of the ribosomal protein S6, which enables enhanced protein synthetic capacity, a prerequisite for cell growth and proliferation [77]. Consistent with this, we observed phospho‐S6 levels generally paralleling changes in phospho‐AKT levels upon IRF4 expression manipulation (Fig. 5B,C), providing further evidence for the positive influence of IRF4 on AKT pathway activity. On the other hand, we observed that ectopic IRF4 overexpression enhanced AKT pathway activity markers also in UACC62 cell line which has homozygous deletion at the *PTEN* locus, precluding PI3K‐AKT activation via IRF4‐mediated repression of PTEN methylation and expression. Upon further examination of the literature, we found the description of an alternative pathway in human melanoma cells and PTEN‐null mouse for AKT pathway activation where DNMT3B represses via promoter methylation mir‐196b, and consequently activates the expression of RICTOR, an mTORC2 component, which is an upstream activator of the AKT pathway [78]. Based on this, we tested the involvement of IRF4 in this pathway by investigating DNA methylation at the promoter of mir‐196b, the central regulator of this pathway. Our results showed that experimental manipulation of IRF4 levels in all the six cell lines tested altered promoter methylation of mir‐196b (Fig. 5D,E; Fig. S5A,B) consistent with the DNMT3B‐mediated methylation observed by Micevic et al. [78]. This provides evidence for an alternative, PTEN independent pathway linking IRF4 to AKT pathway (i.e., via IRF4‐DNMTs‐DNA methylation‐mir196b‐RICTOR/mTORC2‐AKT) in both PTEN‐deficient and ‐sufficient contexts, in addition to the one involving PTEN (promoter methylation) in PTEN‐sufficient contexts.

### 3.6 IRF4 modulates melanoma cell responses to epigenetic drugs

**Fig. 6:**
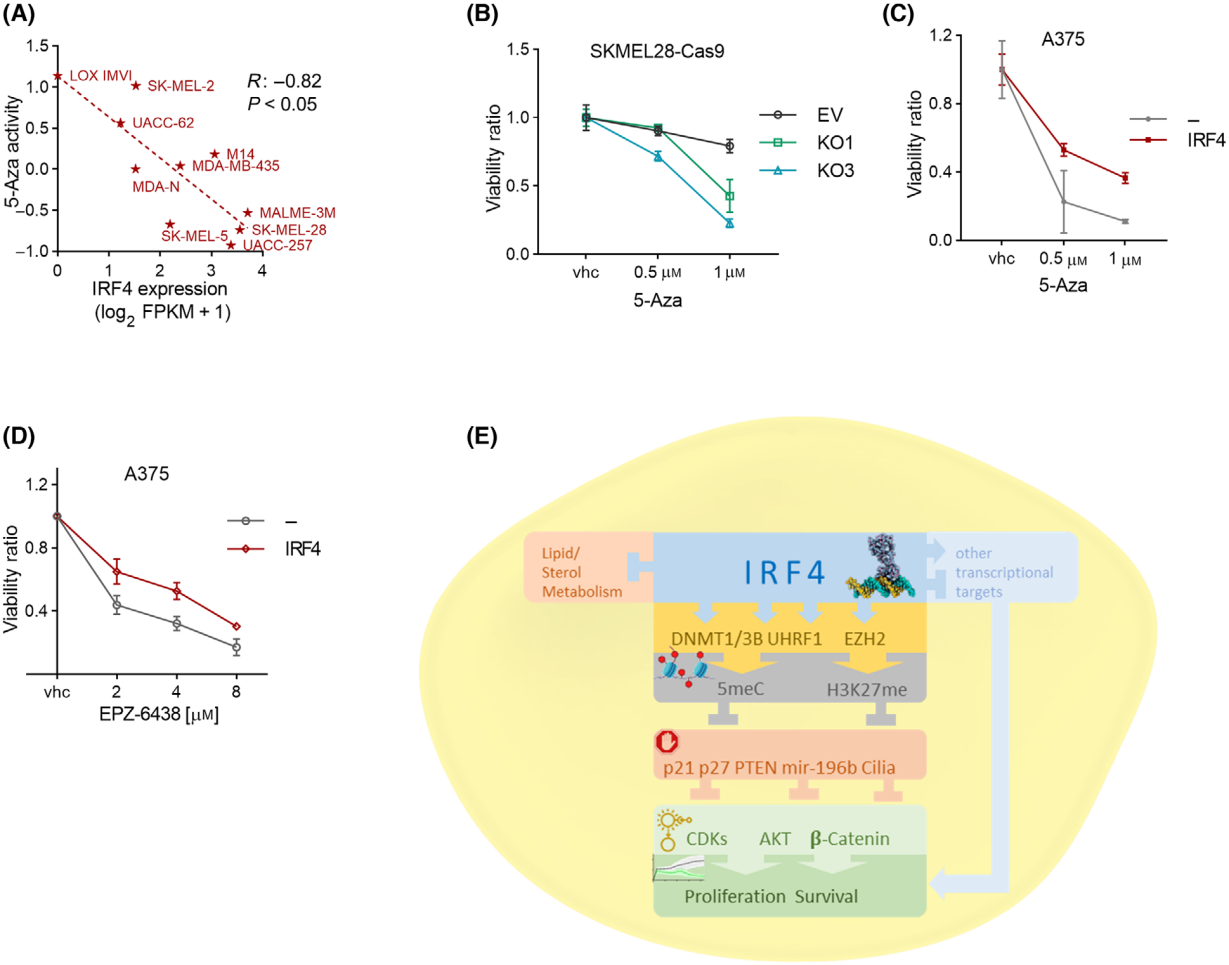
IRF4 modulates melanoma cell responses to epigenetic drugs. (A) Correlation analysis for NCI‐60 melanoma cell line panel azacytidine (5‐Aza) drug activity response *versus* IRF4 mRNA levels. Data retrieved from Cell Miner Database [79]. (B, C) XTT cytotoxicity analysis to assess the effect of 5 days of 5‐Aza treatment (B) with IRF4 depletion; (C) with IRF4 overexpression. (D) XTT analysis to assess the effect of 6 days of EZH2i EPZ‐6438 (Tazemetostat) treatment with IRF4 overexpression. Error bars depict ±SEM from three independent experiments. (E) Working model on the mechanisms of action of IRF4 in melanoma cells based on study findings. Briefly, IRF4 is a dependency factor in melanoma, influencing cell proliferation and survival (dark green layer). This study reveals a number of molecular mechanisms in this process through the regulation of epigenetic silencing factors (yellow layer), cognate molecular marks (grey layer), and downstream tumour suppressors (red layer) and oncogenic pathways (light green layer) known to be functionally linked via previous studies and/or this study. In addition, IRF4 regulates the expression of a large number of genes, products of some of which may modulate the dependency phenotype via pathways that are common/related to those studied here, or independently. Data Information: In (A), Spearman correlation *R* = −0.82, linear regression *P* = 0.0038. In (B), error bars depict SEM from three independent experiments. Statistical analysis with 2‐way ANOVA with Bonferroni's multiple comparisons. The adjusted *P*‐values for KO1 and KO3 are > 0.99 in vhc controls, > 0.99 and 0.09 in 0.5 μm and < 10^−4^ in 1 μm samples, respectively. In (C, D), Welch's *t*‐test was carried out. In (C), the *P*‐values are 0.12 for 0.5 μm and 0.0039 for 1 μm. In (D), the adjusted *P*‐values are 0.0329 for 2 μm, 0.0481 for 4 μm, and 0.4204 for 8 μm. The other *P*‐values are > 0.99.

The dependency factor role of IRF4 in melanoma cells, as well as its function in the regulation of repressive methylations, raised the possibility that it could also modify the response of melanoma cells to the relevant epigenetic inhibitor drugs. Accordingly, we observed in NCI‐60 cell line collection CellMiner CDB data [79] that the cytotoxic efficacy of the DNMTi drug 5‐azacytidine (5‐Aza) was significantly anti‐correlated with IRF4 levels in melanoma cell lines (Fig. 6A), but not in the data set as a whole (Fig. S6A). Therefore, we wanted to see if IRF4 could modulate the sensitivity of melanoma cells to DNMTi. First, we confirmed that DNMTi treatment reduced genome‐wide DNA methylation in melanoma cells (Fig. S6B). Using *in vitro* cytotoxicity assays, we observed that IRF4 depletion sensitised melanoma cells to 5‐Aza treatment (Fig. 6B). We recapitulated these findings with a related but distinct DNMTi, 5‐Aza‐2′‐deoxycytidine (decitabine; DAC; Fig. S6C), providing support for the specificity of the results. Furthermore, IRF4 overexpression partially rescued both Aza‐ and DAC‐mediated cytotoxicity (Fig. 6C; Fig. S6D–F) in line with the upstream regulatory role of IRF4 on DNA methylation. Similarly, IRF4 overexpression partially rescued EZH2i (EPZ‐6438; tazemetostat) mediated cytotoxicity (Fig. 6D; Fig. S6G,H), again in line with the upstream regulatory role of IRF4 on H3K27 methylation.

## 4 Discussion

Studies in melanoma over the past decades have identified several critical genes with tumour‐suppressive and oncogenic functions in diverse processes such as core cell cycle regulation and upstream pathways, most prominently in mitogen activated protein kinase (MAPK), PI3K‐AKT, and WNT/β‐catenin pathways. Despite initial efficacy of therapies based on some of these discoveries and the impressive recent progress in melanoma immunotherapies, late‐stage melanomas are still mostly refractory to therapies and ultimately lethal, highlighting the need for fresh insights into the biology and therapy of melanoma. IRF4 was first established as a developmental regulator in various immune cell types. Since then, we and others have described IRF4 as a dependency factor in lymphocyte‐derived cancers where IRF4 transcriptionally regulates both common and cancer‐specific downstream genes important for survival and/or proliferation. On the other hand, numerous genetic studies over the past two decades linked IRF4 to both melanocyte function and melanoma. While its role in melanocyte function has been substantiated through mechanistic studies, if and how IRF4 is causally involved in melanoma has remained largely obscure. Only recently IRF4 was proposed to regulate the expression of PD‐L1, the ligand for the negative immune regulatory receptor PD‐1, via IRF1 [80] and with ERG1 [81], leading to immune evasion and anti‐PD‐1/PD‐L1 therapy resistance in melanoma. However, melanoma cell‐intrinsic mechanisms of action of IRF4, if any, still remained elusive.

In this study, we first confirm that IRF4 expression is prevalent in melanoma cells. Nevertheless, not all melanoma tumours or cell lines detectably express IRF4. This heterogeneity can at least partially be explained by the status of upstream regulators of IRF4 and by the particular genotypic variation at the functional SNP that regulates the melanocyte‐specific intronic enhancer of *IRF4* via these regulators [15]. In agreement with this, we observe higher IRF4 expression in melanoma cell lines such as SKMEL28, SKMEL5, MALME3M and G361 with the C/C genotype at this SNP (known for higher IRF4 expression in melanocytes), compared to undetectable expression in lines with C/T or T/T genotypes and/or those lacking critical regulators such as MITF which acts through this SNP‐containing enhancer (A375, UACC62). More importantly, we observe that IRF4 expression in melanomas is inversely associated with patient survival, likely due to its dependency factor status in melanoma cells, suggested by our GFP competition assays and the publicly available CRISPR/Cas9 dropout screen data where IRF4 expression tracks dependency scores. In line with our observations, the dependency factor status of IRF4 was also underscored by a recent study, again based on published CRISPR screen data [82] while this manuscript was in preparation. The aforementioned study also highlighted recurrent genomic amplifications spanning *IRF4* locus in melanoma patient tumours as a source of high IRF4 expression [82] which could be yet another contributor to IRF4 expression heterogeneity in melanoma. Therefore, we conclude that while IRF4 expression and dependency are not universal in melanomas, the sizeable proportion of melanomas that express IRF4 has dependency on it, similar to the observations in lymphocyte‐derived cancers. What molecular mechanisms substitute IRF4 activity in melanomas with low/nonexistent IRF4 expression is a question that merits further investigation.

We identified in our RNA‐seq data a previously undescribed category of IRF4‐regulated genes: chromatin modifiers. Due to the novelty of this finding, and the growing appreciation of epigenetic processes in cancer biology and their applications in therapy, we focused on those IRF4‐regulated epigenetic modifications with relatively established and critical roles in melanoma biology, i.e., DNA 5‐cytosine methylation and histone H3 lysine 27 methylation. Since aberrant DNA and H3K27 methylations have oncogenic properties in melanoma, mostly mediated by the overexpression of the relevant enzymes, identification of upstream regulators of these methylations is of interest as a step towards development of novel therapeutic approaches. However, our understanding of such upstream regulators in melanoma, especially direct transcriptional regulators, had been relatively limited. Accordingly, we show that IRF4 transcriptionally regulates in melanoma cells multiple components of the DNA methylation machinery, and is a driver of DNA methylation, a process known to be causally involved in cancer, for example through promoter methylation and silencing of various TSGs. Consequently, we show that IRF4 regulates the promoter methylation and expression of multiple genes with tumour suppressive functions. Among these are the CDK inhibitors that are negative regulators of cell cycle progression, such as p21/Cip1/Waf1 and p27/Kip1. This observation led us to investigate the cell cycle phase profiles of IRF4‐depleted cells, for which we saw moderate but consistent changes that are in line with stalled or slowed down cell cycle progression, and an opposite pattern with IRF4 overexpression. These findings also complement results of experiments with IRF4 depletion showing reduced proliferation/survival and colony forming capacity in this study. On the other hand, although short‐term experimental IRF4 overexpression is achievable (therefore enabling certain experiments), IRF4 overexpression is detrimental to melanoma cell proliferation in long‐haul assays such as real‐time cell analysis and colony formation, where phenotypes partly similar to those with IRF4 depletion are observed (unpublished observations). Although the mechanisms behind these observations are yet to be studied, it can be speculated that processes such as oncogene‐ or differentiation‐induced senescence could lead to the proliferation defects in melanoma cells with excessive levels of IRF4, as observed for other cancer‐critical transcriptional regulators, such as MITF [83].

Our transcriptomic experiments also pointed to the oncogenic histone H3K27 methyltransferase EZH2 as a target of IRF4 transcriptional activation in melanoma cell lines. Since EZH2 is a versatile oncogenic factor with therapeutic targeting potential, we were interested in studying IRF4's role in the regulation of EZH2 and its downstream effectors. After verifying IRF4 regulation of EZH2 expression and showing its consequences on H3K27 methylation, we focused on the relatively recently described EZH2‐primary cilia‐β‐catenin axis [70] in the context of IRF4. Our results showed that the effect of IRF4 indeed extends to primary cilia and to the downstream oncogenic β‐Catenin pathway, at least partly through its effects on EZH2. However, given the extensive breadth of targets that EZH2 modulates in melanoma [63, 72], it is likely that additional EZH2 downstream processes are also relevant for IRF4's observed effects. In this regard, even the *CDKN1A*/p21 TSG that we studied in the context of promoter DNA methylation is known to experience EZH2/H3K27me‐mediated repression in melanoma [84], pointing to potential high‐level connectedness of regulatory mechanisms downstream of IRF4 where overlaps and redundancies among epigenetic silencing mechanisms [85, 86] at times limit the definition of exclusive linear pathways.

The PI3K‐AKT signalling pathway is another pathway that frequently plays oncogenic roles in diverse cancers, including melanoma, where somatic loss‐of‐function or silencing of the negative regulator *PTEN* is an occasional driver [87, 88]. Our results implicating IRF4 as a regulator of *PTEN* promoter methylation and silencing prompted us to make a limited foray into its downstream effects, where we observed modulation of phosphorylation (and presumably, of activity) in the anabolic AKT‐S6K‐S6 axis by IRF4. These results are in line with our observations of decreased proliferation upon IRF4 depletion. Whether these observations can be supplementary to other pathway axes downstream of AKT, such as cell survival or metabolism, requires further research.

Therefore, in this study we probed several known cancer‐critical pathways downstream of epigenetic silencing and observed their IRF4 dependence. This multiplicity of downstream cancer‐critical effector pathways emanating from a cancer dependency factor is not uncommon, especially in the case of transcription factors, which typically rank high in regulatory hierarchies, and have numerous targets, leading to pleiotropic fitness deficits upon their removal or dysregulation (e.g., c‐Myc in various cancers [89]; MITF in melanoma [83]). Hence, the melanoma‐critical downstream target pathways of IRF4 investigated in this study are likely not exhaustive, as also hinted by the large number of DEGs identified by our transcriptomic study. Therefore, while most of our work here highlights pathways that IRF4 regulates proliferation through epigenetic silencing of TSGs and consequent activation of oncogenic pathways, this may not be the whole story. For instance, mitotic cell division cycle and/or proliferation‐related ontologies are prominently enriched in the common IRF4‐activated DEG set (Fig. S2A), exhibiting numerous genes whose products are involved in the structural, catalytic or regulatory aspects of cell proliferation (Table S4), such as those associated with DNA replication and repair (e.g., *POLE*, *POLG*, *RPA2*, *RFC5*, *TOP2A*), core cell cycle regulatory machinery (e.g., *CCNA2*, *CCNB1*, *CCND3*, *CCNE1*, *CDK1*), and chromosome segregation (e.g., *CENPE*, *CENPF*, *CENPW*, *BUB1*, *BUB3*, *CDC20*, *MAD2L1*, *AURKA*, *PLK1*).

We therefore surmise a two‐pronged strategy used by IRF4 to promote melanoma proliferation: direct transcriptional regulatory routes which bulk up the required proliferation machinery, and more circumvent paths through epigenetic silencing, exerting regulation via signalling pathways (Fig. 6E). On the other hand, we cannot rule out that the former also directly contributes to proliferation, since mere overexpression of certain cell cycle regulatory components is known to have proliferative and oncogenic outcomes under appropriate conditions, such as in the case for cyclin D [90].

Nevertheless, given growing reach of IRF4‐targeting strategies for cancer therapy via IMiDs [91] and antisense oligonucleotides [10, 92] in blood cancers, we believe that further research on targeting IRF4 in melanoma is justified, for instance using *in vivo* models. IRF4‐targeting approaches may provide particular advantages over inhibiting individual downstream methyltransferases. Since IRF4 controls both DNA methylation and H3K27me, inhibiting IRF4 is expected to prevent any potential redundancy among these gene repressive mechanisms [85, 86]. Nonetheless, based on our sensitisation results with IRF4 and methylation co‐targeting, further work on combination approaches may also show promise. The influence of IRF4 on the efficacy of these epi‐drugs, combined with the association of the corresponding *cis*‐regulatory SNP and copy number variation to IRF4 expression heterogeneity, can be expected to provide a handle for personalisation of any potential IRF4‐ and methyltransferase inhibitor‐directed therapeutic strategies in melanoma. Moreover, beyond the cancer cell‐intrinsic anti‐tumour activities of DNA and histone methyltransferase inhibitors, there's a growing appreciation for the tumour immunity‐modulating effects of epigenetic inhibitors, such as sensitisation to immune checkpoint inhibitor therapy [93]. This concept dovetails well with the recent reports demonstrating immune(therapy)‐modulating activities of IRF4 in melanoma [80, 81], and therefore merits further investigation.

## 5 Conclusions

The involvement of IRF4 in melanoma has long been known through expression and genetic association studies. Although recent studies showed IRF4's involvement in melanoma‐host immune system interactions, mechanistic understanding into IRF4's melanoma‐intrinsic role has been lacking.

Consequently, in this study, we not only confirm IRF4 as a dependency factor for melanoma cells but also show that it modulates epigenetic silencing and repression of tumour suppressors, impinging on critical oncogenic pathways such as WNT/β‐catenin and AKT (Fig. 6E). These mechanistic insights are expected to inspire further studies for targeting IRF4‐expressing melanomas.

## Conflict of interest

The authors declare no conflict of interest.

## Author contributions

US contributed to conceptualization, formal analysis, investigation, methodology, supervision, visualisation, writing – original draft, writing – review & editing; BÇ contributed to investigation; EY contributed to formal analysis, investigation, methodology; NYA, LH, MCA, CY and HKG contributed to investigation, methodology; YBA contributed to investigation; ENF‐K contributed to methodology, resources, supervision; NCTE contributed to conceptualization, formal analysis, funding acquisition, investigation, project administration, supervision, visualisation, writing – original draft, writing – review & editing.

### Peer review

The peer review history for this article is available at https://www.webofscience.com/api/gateway/wos/peer‐review/10.1002/1878‐0261.13672.

## Supporting information


**Fig. S1.** IRF4 expression is common in melanoma and is associated with dependency and poor patient survival.
**Fig. S2.** IRF4 modulates DNA and histone H3 Lysine 27 methylations in melanoma cells.
**Fig. S3.** IRF4 regulates multiple melanoma‐critical tumour suppressor genes and the cell cycle.
**Fig. S4.** IRF4 is an upstream regulator of WNT/β‐catenin pathway in melanoma cells.
**Fig. S5.** IRF4 is an upstream regulator of AKT pathway in melanoma cells.
**Fig. S6.** IRF4 modulates melanoma cell responses to epigenetic drugs.

**Table S1.** shRNA & sgRNA sequences.
**Table S2.** Antibodies.
**Table S3.** Oligonucleotide primers.
**Table S4.** IRF4‐activated genes.
**Table S5.** IRF4‐repressed genes.
**Table S6.** IRF4‐activated GO terms.
**Table S7.** IRF4‐repressed GO terms.
**Table S8.** log2‐fold expression changes of the genes in the Reactome “Epigenetic regulation of gene expression” Pathway.

## Data Availability

RNA‐seq data are available at Gene Expression Omnibus (GEO; RRID: SCR_005012) with the accession no. GSE244460. The data that support the findings of this study are available from the corresponding author upon reasonable request.
