# Supplementary material for: Interferon regulatory factor 4 modulates epigenetic silencing and cancer‐critical pathways in melanoma cells
Source: Mol Oncol. 2024 Jun 16;18(10):2423–48. doi: 10.1002/1878-0261.13672 (PMC11459048; doi:10.1002/1878-0261.13672)
Supplement: Supplementary file 1 — Fig. S1. IRF4 expression is common in melanoma and is associated with dependency and poor patient survival. Fig. S2. IRF4 modulates DNA and histone H3 Lysine 27 methylations in melanoma cells. Fig. S3. IRF4 regulates multiple melanoma‐critical tumour suppressor genes and the cell cycle. Fig. S4. IRF4 is an upstream regulator of WNT/β‐catenin pathway in melanoma cells. Fig. S5. IRF4 is an upstream regulator of AKT pathway in melanoma cells. Fig. S6. IRF4 modulates melanoma cell responses to epigenetic drugs. [file MOL2-18-2423-s001.zip › FigsS1-S6_Legends.docx]

**LEGENDS FOR SUPPLEMENTARY FIGURES**

**Figure S1. IRF4 expression is common in melanoma and is associated with dependency and poor patient survival. A)** Subcellular localization of IRF4 in Immunofluorescence staining (IF) of G361 melanoma cell line with Alexa 647 conjugated anti-IRF4 antibody and DAPI. HEK293FT and MCF-7 used as negative controls. Secondary antibody-only staining carried out to ensure undetectable background staining. Scalebar: 50 µm. **B)** IRF4 protein expression in G361 cell lines with subcellular fractionation followed by immunoblotting. GAPDH used as a cytoplasmic fraction (‘CF’) marker, whereas histone H3 used as a nuclear fraction (‘NF’) marker. **C)** IRF4 mRNA expression in the Cancer Cell Line Encyclopedia (CCLE) RNA-seq datasets. Blue: lymphoid cancers, red: melanoma; gray: other cancers. FPKM: log-scale fragments per kilobases per million mapped reads. **D)** Overall survival (in days) Kaplan-Meier curves for melanoma patients with IRF4^high^ (red, top quartile, n=12) and IRF4^low^ (blue, bottom quartile, n=12) groups in Bogunovic et al. 2009 microarray data (GSE19234). p=0.042 by log-rank (Mantel-Cox) test. **E)** Correlation analysis of IRF4 expression (via RNA-seq) *versus* dependency (via CRISPR KO dropout screening) data in melanoma cell lines. Lower gene effect (Chronos) scores indicate a higher likelihood that a given gene is essential in a given cell line. A score of 0 or above indicates that a gene is not essential (Pacini et al., 2021). Spearman correlation coefficient R = -0.70, linear regression p < 10^-4^. **F)** Assessment of competitive fitness of G361 cells with IRF4 depleted *versus* control cells in GFP competition assay. Error bars depict SEM from 2 independent experiments. The two-way ANOVA with multiple comparisons using the Dunnet method was performed. The adjusted p-value for shIRF4 vs. shLuc is <10^-4^ for days 10, 12, and 14, and for day 7, 0.0046 and >0.99 for other time points. **G)** Real-time cell analysis with the XCELLigence® instrument to gauge the effect of cell accumulation in IRF4-depleted melanoma cells. Error bars shown as area fill.  **H)** Trypan blue exclusion assay results in IRF4 knocked-out SKMEL28-Cas9 cells at day 15, with two independent guides (KO1 and KO3). Error bars depict SEM from 3 independent experiments. P-values calculated using Welch’s t-test. The p-value for KO1 and KO3 samples are 0.0038 and 0.0024 respectively.

**Figure S2. IRF4 modulates DNA and histone H3 Lysine 27 methylations in melanoma cells.** Biological Process gene ontology enrichment analysis with GOrilla (Eden et al, 2009) for common DEGs in SKMEL28 and SKMEL5 cells (i.e, common set of DEGs significant at false discovery rate q < 0.001): **A)** for IRF4-activated DEGs (inset highlights epigenetics-related processes), **B)** for IRF4-repressed DEGs. Illustrations created by GOrilla web server (https://cbl-gorilla.cs.technion.ac.il/). **C)** RT-qPCR mRNA expression analysis of DNMT1, DNMT3B, UHRF1, and EZH2 in a panel of cell lines. Top three rows: with IRF4 knockdown; bottom three rows: with IRF4 overexpression. Housekeeping gene RPS28 normalized average signals, further normalized to the corresponding control samples, set at 100. Error bars depict SEM from 3 technical replicates. Representative data from at least 2 independent experiments shown. **D)** Western blot analysis of IRF4 overexpression in SKMEL28 cell line for DNMT1, DNMT3B, UHRF1, and EZH2 levels. **E)** Western blot analysis of DNMT1, UHRF1 and EZH2 in lentivirally delivered doxycycline-inducible IRF4 knockdown G361 cell lines on day 5(D5) of knockdown. **F)** Western blot analysis of IRF4 knockout samples for EZH2 protein levels. **G)** Western blot analysis of histone H3 lysine 4 monomethylation (K4me1) with IRF4 knockdown. Total histone H3 and GAPDH are loading controls.

**Figure S3. IRF4 regulates multiple melanoma-critical tumor suppressor genes and the cell cycle. A)** RT-qPCR of PTEN, p21 and p27 genes in IRF4 overexpressing MELST cells. **B)** Immunoblot analysis of PTEN, p21, and p27 expression in IRF4 knockdown G361 melanoma cell line. **C-D)** Immunoblot analysis of PTEN, p27, and p21 expression at days 2 and 3 of IRF4 overexpression in **C)** MELST and **D)** SKMEL28 cell line. **E)** RT-qPCR analysis of p21, p27, and PTEN expression in DNMT inhibitor decitabine (Dac)-treated cells. **F)** MSRE-qPCR analysis of promoter methylation levels at *PTEN*, *CDKN1A* (encoding p21), and *CDKN1B* (encoding p27) in IRF4 overexpressing MELST cell line. **G-H)** qPCR analyses of restriction enzyme undigested DNA to ensure comparable input gDNA **G)** with IRF4 knockdown and **H)** with IRF4 overexpression. **I)** RT-qPCR analysis of PTEN, p21 and p27 genes in cells with doxycycline-induced IRF4 overexpression and Decitabine (Dac) treatment in MELST cell line, similar to Fig. 3G. **J)** Propidium iodide (PI)-based flow-cytometric measurement of cell cycle phases in IRF4 overexpressing SKMEL28 cell line.

Data Information: Error bars depict SEM from 3 (A, B, G) and 2 (E, H, I) independent experiments. In (A, B, E), Welch’s t-test was performed. In (A, B), p-values for PTEN, p21 and p27 are 0.0002, 0.001, 0.0009 in G361 and 0.00018, 0.0011, 0.0005 in MELST, respectively. In (E), p-values for p21, p27 and PTEN are 0.046, 0.043, 0.033 in 1 µM respectively. In (F), p-values for PTEN, CDKN1A and CDKN1B are 0.0213, 0.0114, 0.0246 respectively. The p-value for PTEN in 0.5 µM is 0.030. In (I), p-values are 0.0254 for PTEN and 0.0377 for p27. The p-value for PTEN in 0.5 µM is 0.030. In (J), two-way ANOVA with Bonferroni’s multiple comparison tests for changes in cell cycle phases between samples depicts p-value of 0.023, and 0.0076 in SKMEL28 and MELST respectively.

**Figure S4. IRF4 is an upstream regulator of WNT/β-catenin pathway in melanoma cells. A)** RT-qPCR for 3 cilia genes, WDR19, TULP3 and WDR34 in doxycycline-inducible EZH2 overexpressing SKMEL28 cells. **B-C)** ChIP-qPCR with anti-H3K27me3 antibody at *WDR19* locus: **B)** H3K27me3 enrichment at *WDR19* locus; Error bars depict SEM of 2 (MALME3M) and 3 (SKMEL28) biological replicates **C)** the effect of IRF4 knockout (SKMEL-28 Cas9 cells) or IRF4 knockdown (MALME3M) on H3K27me3 signal at *WDR19* locus. Error bars depict SEM of 2 biological replicates. **D) )** ChIP-qPCR with anti-H3K27me3 antibody at *WDR19* locus in dox-inducible IRF4 overexpressing A375 and MELST cell lines. ACTB promoter is used as negative control. **E)** RT-qPCR analysis of WDR19 expression in IRF4 knockdown cells. Error bars depict SEM of 3 independent experiments. **F)** Immunoblot for WDR19 upon IRF4 knockdown. Red arrow showing main WDR19 band. **G)** RT-qPCR for two other cilia genes, TULP3 and WDR34 in IRF4 depleted SKMEL29 cell lines. **H)** RT-qPCR rescue experiments for 3 cilia genes, WDR19, TULP3 and WDR34 in EZH2 overexpressing (OE) SKMEL28 cells in which IRF4 has been depleted. **I)** Representative image of IF staining of cilia with ARL13b (red) and acetylated tubulin (AcTub; green) in A375. **J-K)** Representative images of IF staining of cilia with ARL13b (red) in cells treated with EZHi (EPZ-6438) for 6 days, without or with IRF4 overexpression. **L)** Quantification of cilia counts in F-G. Error bars depict SEM of 3 technical replicates. Each of the replicates treated with doxycycline and EPZ-6438 independently.

Data Information: In (A-H and L), Welch’s t-test was performed. In (B). p-values for WDR19 are 0.005 in MALME3M and 0.0014 in SKMEL28. In (C), p-values for WDR19 locus are 0.0107 in SKMEL28-Cas9 and 0.0179 in MALME3M. In (E), p-values for WDR19 expression in SKMEL28 and MALME3M are 0.0065, and 0.035 respectively. In (L), p-values for DMSO and EPZ-6438 in IRF4 overexpressing vs control are 0.0014, and 0.0008 respectively. The p-value for IRF4 overexpression in EPZ-6438 vs. DMSO is 0.0045.

**Figure S5. IRF4 is an upstream regulator of AKT pathway in melanoma cells. A-B)** qPCR analyses of restriction enzyme undigested DNA to ensure comparable input gDNA **A)** with IRF4 knockdown and **B)** with IRF4 overexpression.

**Figure S6. IRF4 modulates melanoma cell responses to epigenetic drugs. A)** Correlation analysis for the full NCI-60 cell line panel azacytidine (5-Aza) drug activity *versus* IRF4 mRNA levels. R = -0.04, linear regression p = 0.76. **B)** 5-mC ELISA assay to assess global DNA methylation levels upon treatment with the DNA methylation inhibitor decitabine (DAC) for 3 days. Error bars depict SEM from 2 independent experiments. P-values were calculated by performing two-way ANOVA with Bonferroni’s correction for multiple comparisons. The p-values are for 0.5 µM vs control in A375 and SKMEL28 are 0.0025, 0.0003 respectively. For 1 µM vs control, the p-values are 0.0014 for A375 and 0.0002 for SKMEL28.  **C-H)** XTT cytotoxicity analyses to assess the combined effect of methylation inhibitors with IRF4 expression manipulation: **C)** IRF4 depletion combined with decitabine treatment (4 days); **D)** IRF4 overexpression combined with 5-Aza treatment (5 days); **E- F)** IRF4 overexpression combined with decitabine treatment (4 days); **G-H)** IRF4 overexpression combined with EPZ-6438 treatment (7 days). Error bars depict SEM from at least 2 independent experiments.
