# Supplementary figures and images for: Interferon regulatory factor 4 modulates epigenetic silencing and cancer‐critical pathways in melanoma cells
Source: Mol Oncol. 2024 Jun 16;18(10):2423–48. doi: 10.1002/1878-0261.13672 (PMC11459048; doi:10.1002/1878-0261.13672)

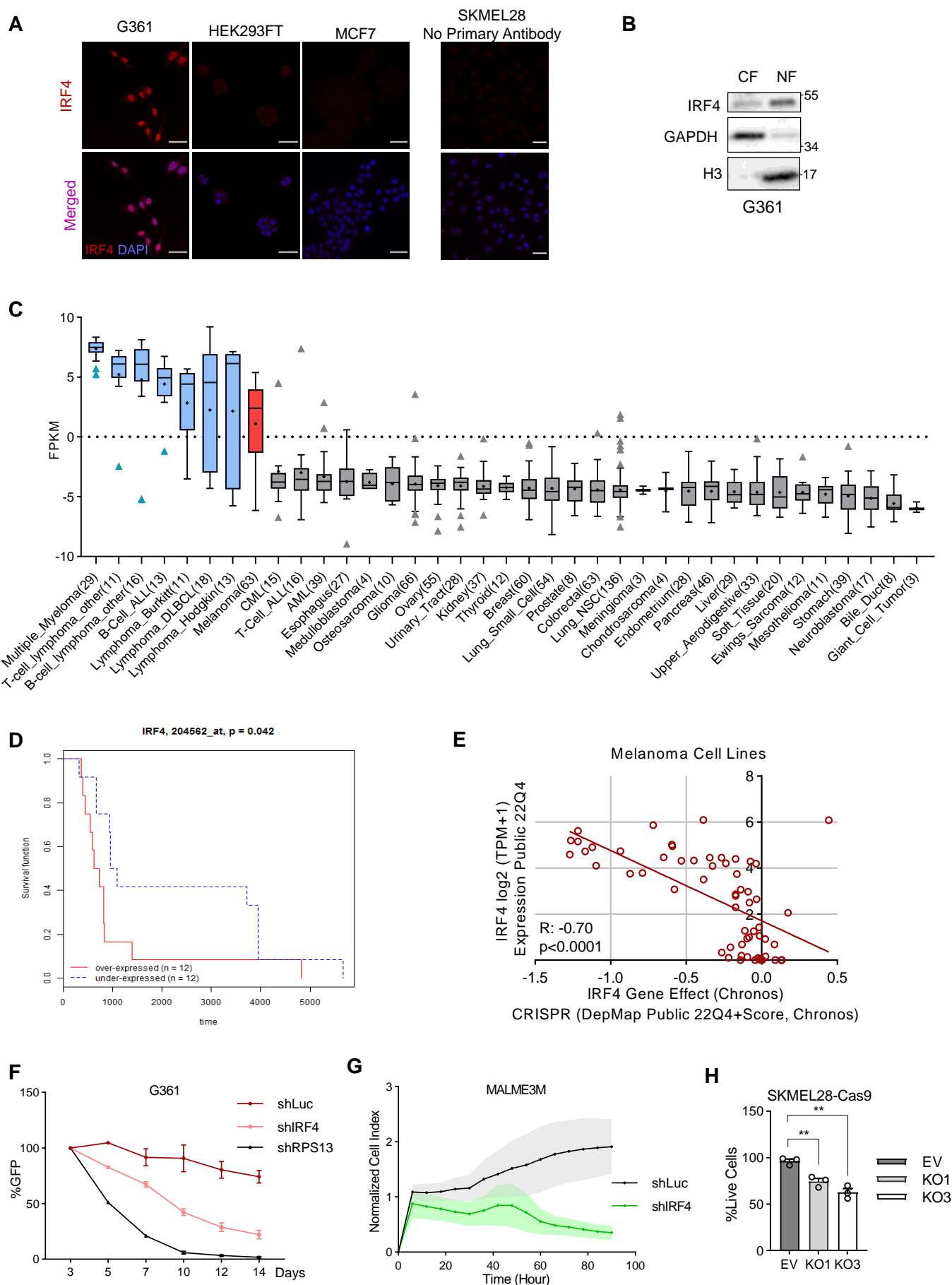

**Figure S1**

**A**

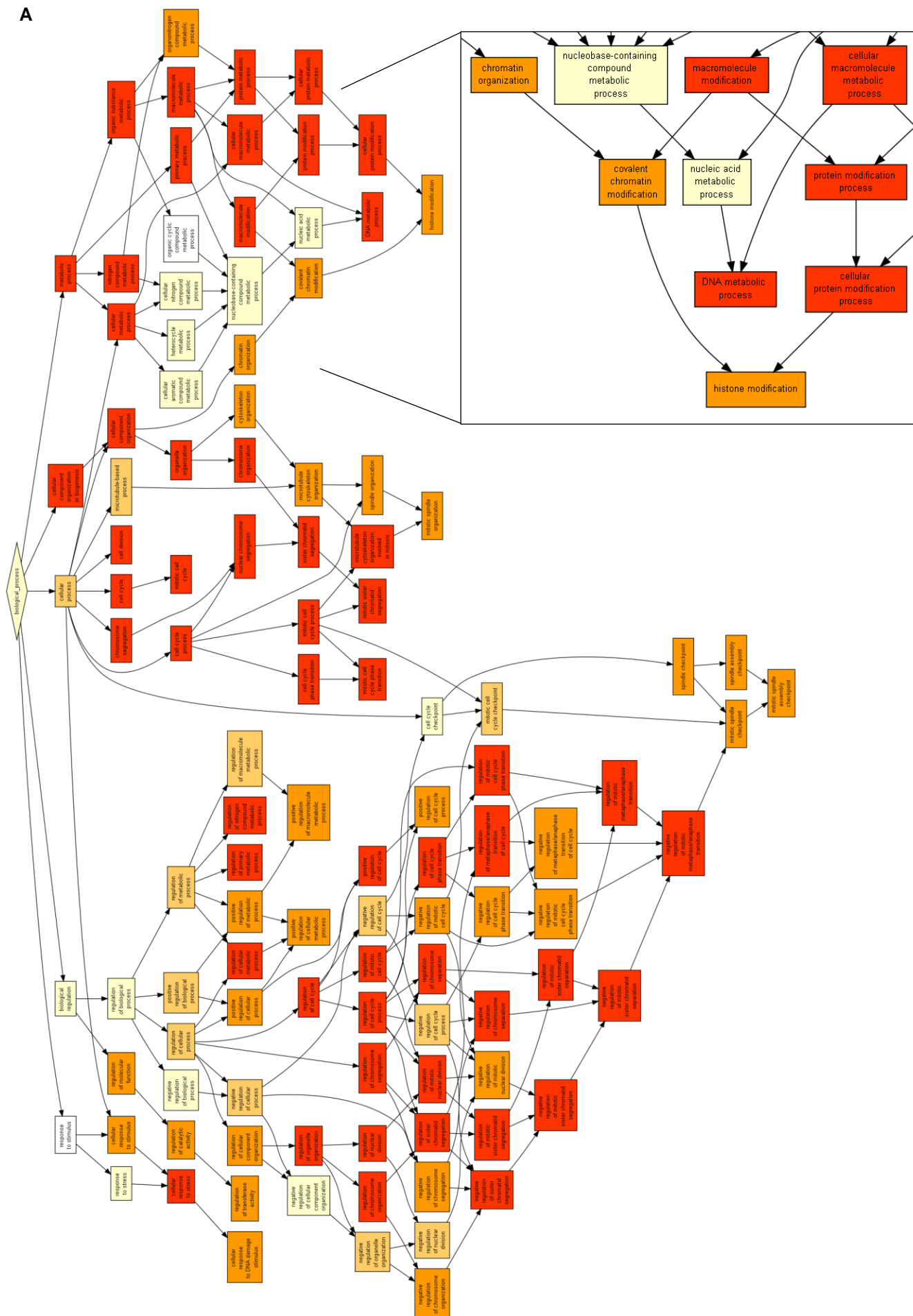

## Figure S2

B

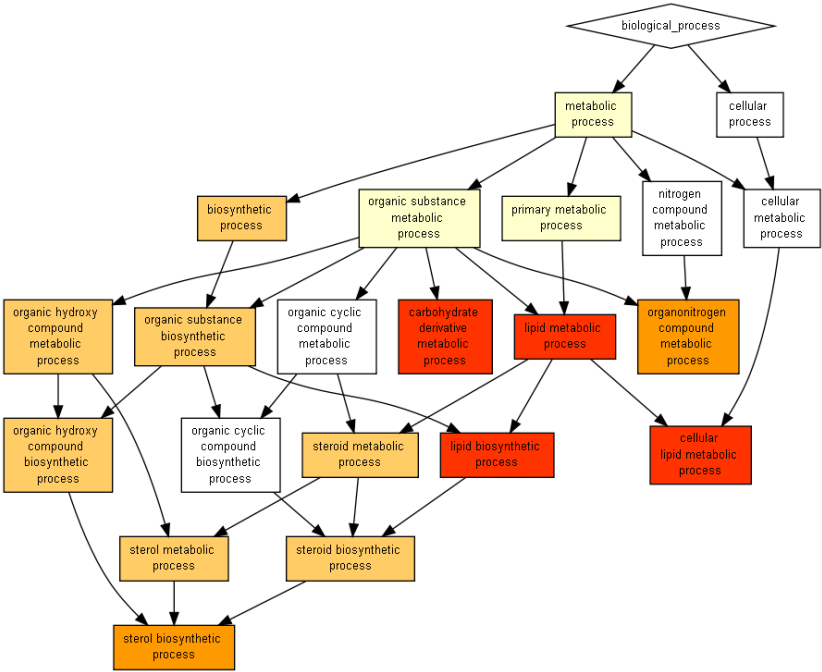

C

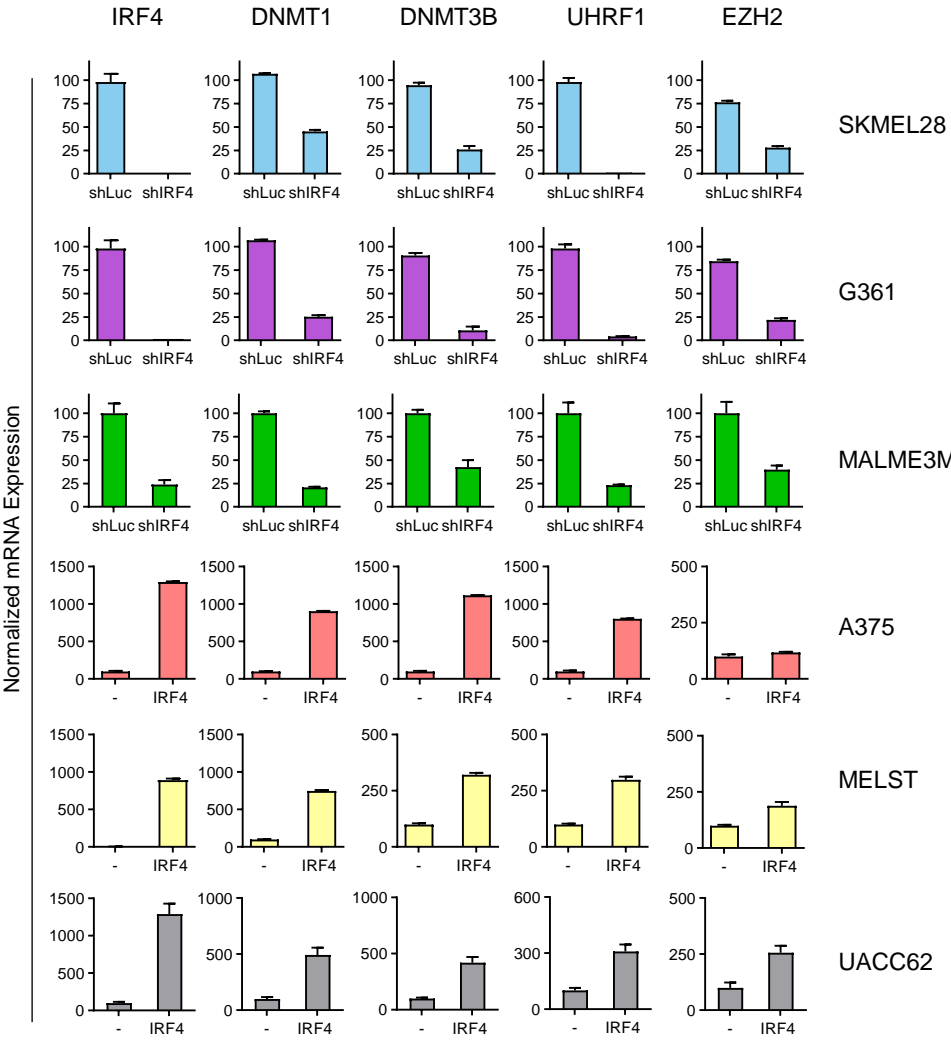

D

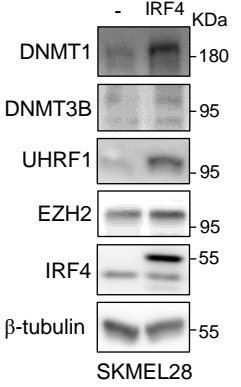

E

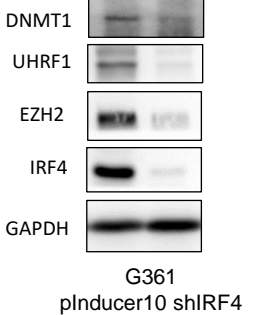

F

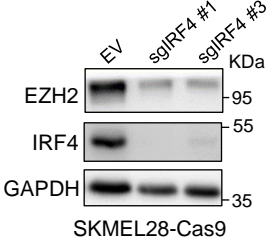

G

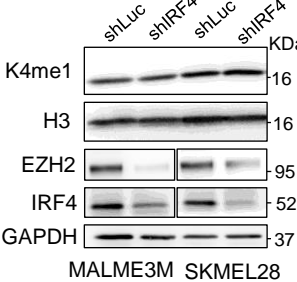

Figure S2

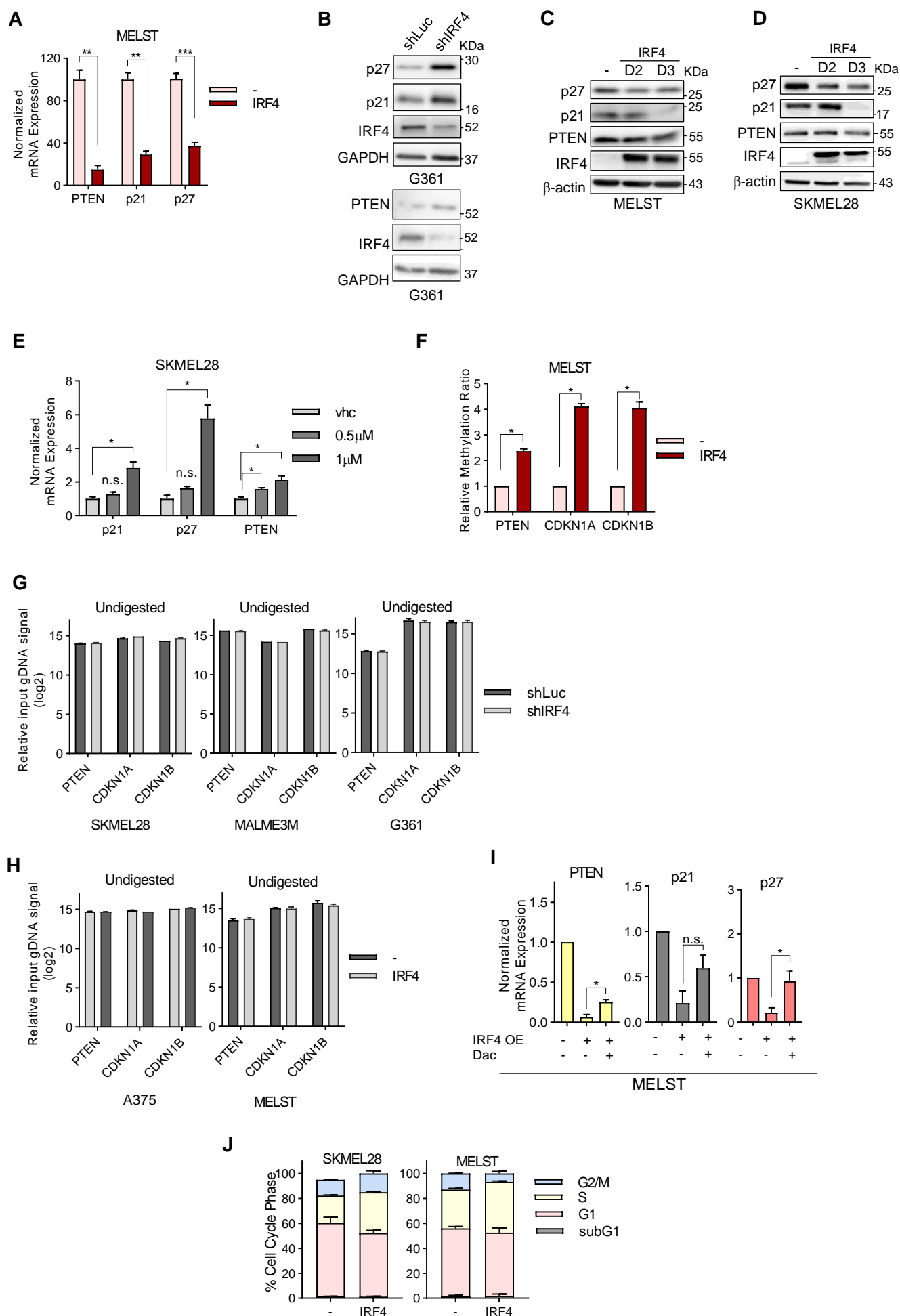

**Figure S3**

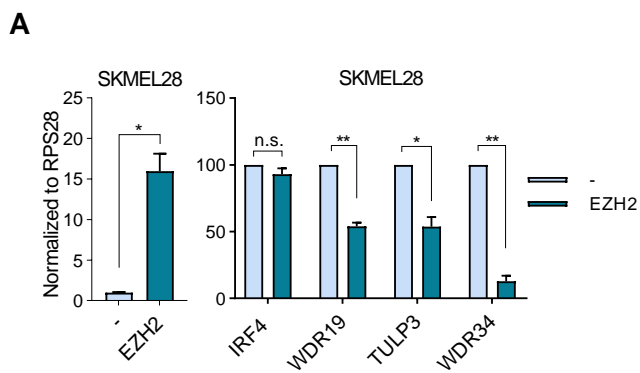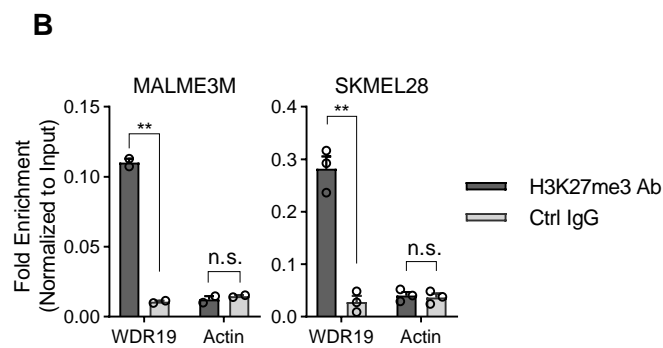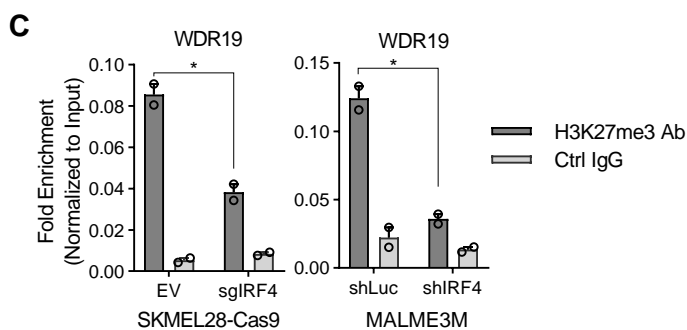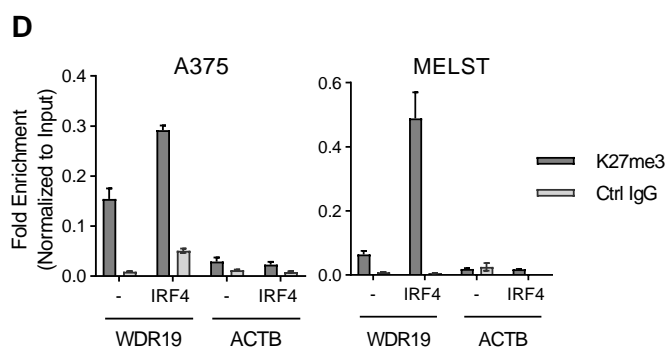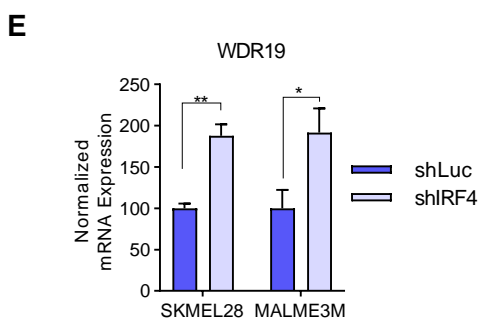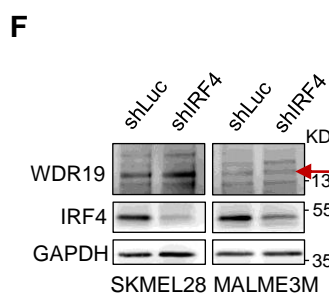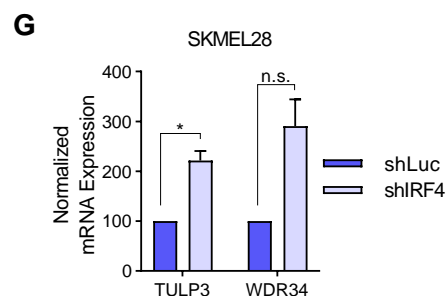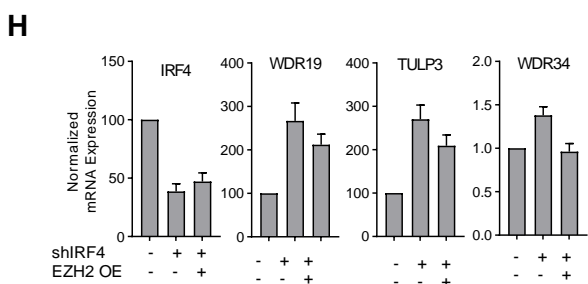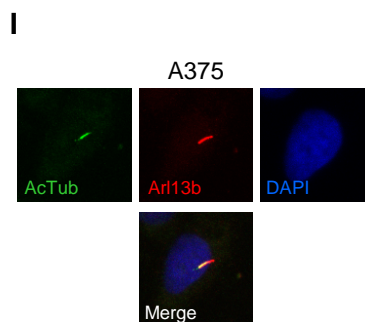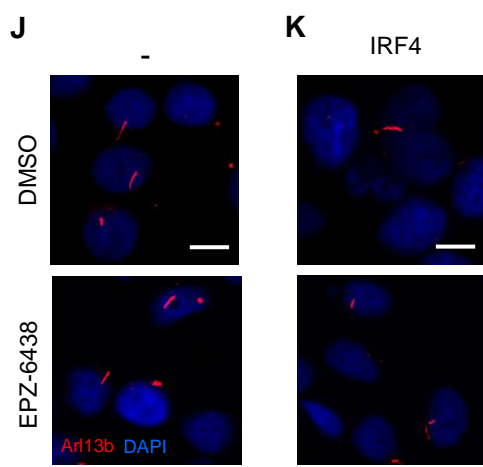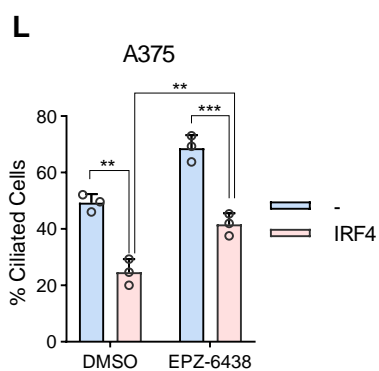

**Figure S4**

A375

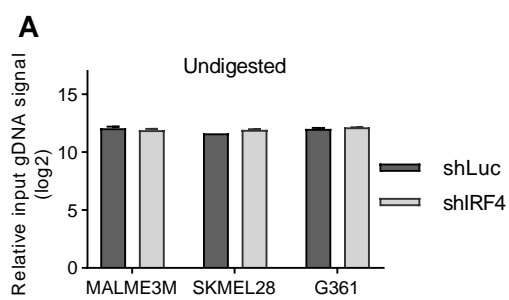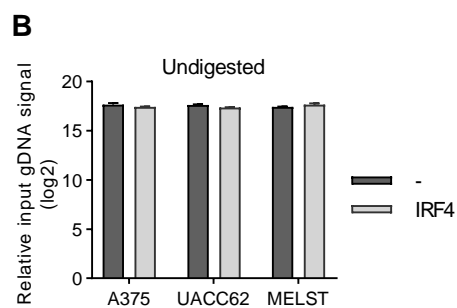

**Figure S5**

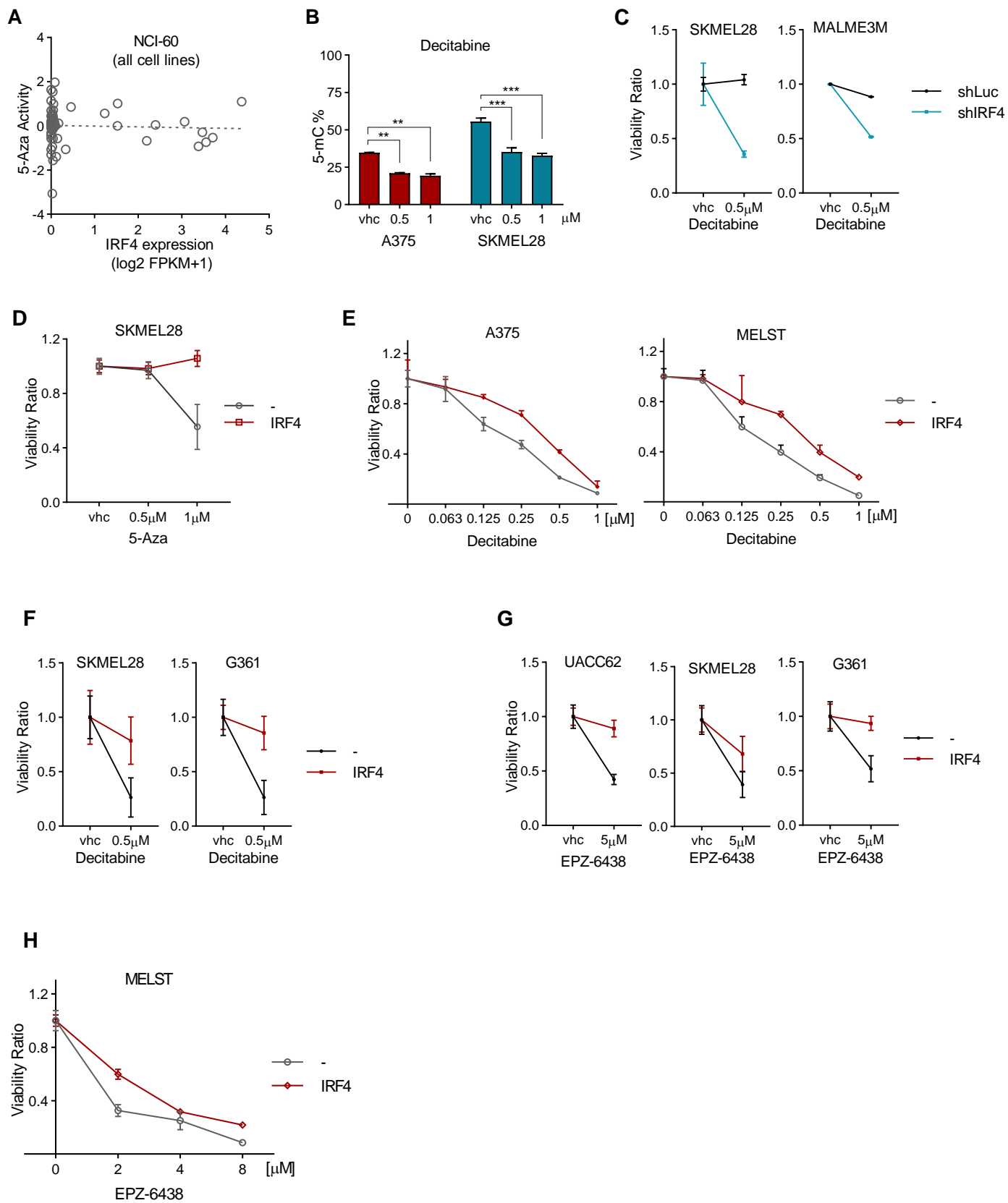

**Figure S6**

Supplement: Supplementary file 1 — Fig. S1. IRF4 expression is common in melanoma and is associated with dependency and poor patient survival. Fig. S2. IRF4 modulates DNA and histone H3 Lysine 27 methylations in melanoma cells. Fig. S3. IRF4 regulates multiple melanoma‐critical tumour suppressor genes and the cell cycle. Fig. S4. IRF4 is an upstream regulator of WNT/β‐catenin pathway in melanoma cells. Fig. S5. IRF4 is an upstream regulator of AKT pathway in melanoma cells. Fig. S6. IRF4 modulates melanoma cell responses to epigenetic drugs. [file MOL2-18-2423-s001.zip › mol213672-sup-0001-FigsS1-S6.pdf]
